# Cooperative Dynamic Manipulation of Unknown Flexible Objects: Joint Energy Injection Based on Simple Pendulum Fundamental Dynamics

**DOI:** 10.1007/s12369-017-0415-x

**Published:** 2017-06-12

**Authors:** Philine Donner, Franz Christange, Jing Lu, Martin Buss

**Affiliations:** 1 Department of Electrical and Computer Engineering, Chair of Automatic Control Engineering (LSR), Technical University of Munich, Munich, Germany; 2 Institute for Advanced Study, Technical University of Munich, Munich, Germany; 3 Department of Electrical and Computer Engineering, Chair of Renewable and Sustainable Energy Systems (ENS), Technical University of Munich, Munich, Germany

**Keywords:** Physical human–robot interaction, Cooperative manipulators, Adaptive control, Dynamics, Haptics, Intention estimation

## Abstract

Cooperative dynamic manipulation enlarges the manipulation repertoire of human–robot teams. By means of synchronized swinging motion, a human and a robot can continuously inject energy into a bulky and flexible object in order to place it onto an elevated location and outside the partners’ workspace. Here, we design leader and follower controllers based on the fundamental dynamics of simple pendulums and show that these controllers can regulate the swing energy contained in unknown objects. We consider a complex pendulum-like object controlled via acceleration, and an “arm—flexible object—arm” system controlled via shoulder torque. The derived fundamental dynamics of the desired closed-loop simple pendulum behavior are similar for both systems. We limit the information available to the robotic agent about the state of the object and the partner’s intention to the forces measured at its interaction point. In contrast to a leader, a follower does not know the desired energy level and imitates the leader’s energy flow to actively contribute to the task. Experiments with a robotic manipulator and real objects show the efficacy of our approach for human–robot dynamic cooperative object manipulation.

**Electronic supplementary material:**

The online version of this article (doi:10.1007/s12369-017-0415-x) contains supplementary material, which is available to authorized users.

## Introduction

**Fig. 1:**
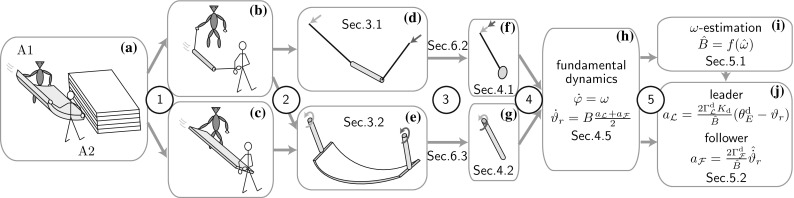
Approach overview: (*1*) Interpretation of flexible object swinging as a combination of pendulum swinging and rigid object swinging. (*2*) Approximation of pendulum swinging by the t-pendulum with 1D acceleration inputs and of flexible object swinging by the afa-system with 1D torque inputs. (*3*) Projection of the t-pendulum and the afa-system onto the abstract cart-pendulum and abstract torque-pendulum, respectively. (*4*) Extraction of the closed-loop fundamental dynamics. (*5*) Fundamental dynamics-based natural frequency estimation and leader and follower controller design

Continuous energy injection during synchronized swinging motion enables a human and a robot to lift a bulky flexible object together onto an elevated location. This example scenario is illustrated in Fig. 1a and combines the advantages of *cooperative* and *dynamic* manipulation. *Cooperative* manipulation allows for the manipulation of heavier and bulkier objects than one agent could manipulate on its own. A commonly addressed physical human–robot collaboration scenario is, e.g., cooperative transport of rigid bulky objects [44]. Such object transport tasks are performed by kinematic manipulation, i.e., the rigid object is rigidly grasped by the manipulators [32]. In contrast, *dynamic* object manipulation makes use of the object dynamics, with the advantage of an increased manipulation repertoire: simpler end effectors can handle a greater variety of objects faster and outside the workspace of the manipulator. Dynamic manipulation examples are juggling, throwing, catching [29] as well as the manipulation of underactuated mechanisms [8], such as the flexible and the pendulum-like objects in Fig. 1a, b.

In this article, we take a first step towards combining the advantages of cooperative and dynamic object manipulation by investigating cooperative swinging of underactuated objects. The swinging motion naturally synchronizes the motion of the cooperating agents. Energy can be injected in a favorable arm configuration for a human interaction partner (stretched arm) and task effort can be shared among the agents. Moreover, the accessible workspace of the human arm and robotic manipulator is increased by the swinging motion of the object and by a possible subsequent throwing phase. In order to approach the complex task of cooperative flexible object swinging in Fig. 1a, we split it up into its two extremes, which are swinging of pendulum-like objects which oscillate themselves (b) and swinging of rigid objects, where the agents’ arms together with the rigid object form an oscillating entity (c). In our initial work, we treated pendulum-like object swinging [13] based on the assumption that all system parameters are known. This assumption was alleviated in [14] by an adaptive approach.

The contribution of this work is three-fold: firstly, we experimentally verify the adaptive approach presented in [14]. Secondly, we combine our results from cooperative swinging of pendulum-like objects and human–human swinging of rigid objects in [15], towards cooperative swinging of flexible objects. Our third contribution lies in the unified presentation of modeling the desired oscillation of pendulum-like and flexible objects through simple pendulum abstractions of equal fundamental dynamics (see two paths in Fig. 1). In the following, we discuss the state of the art related to different aspects of our proposed control approach.

### Dynamic Manipulation in Physical Human–Robot Interaction

Consideration and exploitation of the mutual influence is of great importance when designing controllers for natural human–robot interaction [45]; even more when the agents are in physical contact. Only little work exists on *cooperative dynamic object manipulation* in general, and in the context of human–robot interaction in particular. In [25] and [30], a human and a robot perform rope turning. For both cases, a stable rope turning motion had to be established by the human before the robot was able to contribute to sustaining it. The human–robot cooperative sawing task considered in [38] requires adaptation on motion as well as on stiffness level in order to cope with the challenging saw-environment interaction dynamics.

In contrast, *cooperative kinematic manipulation* of a common object by a human and a robot has seen great interest. Kosuge et al. [26] designed first rather passive gravity compensators, which have been developed further to robotic partners who actively contribute to the task, e.g., [33]. Active contribution comes with own plans and thus own intentions, which have to be communicated and negotiated. Whereas verbal communication allows humans to easily exchange information, human–human studies have shown that haptic coupling through an object serves as a powerful and fast *haptic communication channel* [21]. In this work, the robotic agent is limited to measurements of its own applied force and torque. Thus, the robot has to use the haptic communication channel to infer both, the intention of the partner and the state of the object.

Cooperation of several agents allows for *role allocation*. Human–human studies in [40] showed that humans tend to specialize during haptic interaction tasks and motivated the design of follower and leader behavior [17]. Mörtl et al.  [34] assigned effort roles that specify how effort is shared in redundant task directions. Also, the swing-up task under consideration allows for effort sharing. In kinematic physical interaction tasks, the interaction forces are commonly used for intention recognition, e.g., counteracting forces are interpreted as disagreement [20, 34]. Furthermore, the leader’s intention is mostly reflected in a planned trajectory. For the swing-up task, on the contrary, the leader’s intention is reflected in a desired object energy, which is unknown to the follower agent. Dynamic motion as well as a reduced coupling of the agents through the flexible or even pendulum-like object prohibit a direct mapping from interaction force to intention. We propose a follower that monitors and imitates the energy flow to the object in order to actively contribute to the task.

### Simple Pendulum Approximation for Modeling and Control

The *pendulum-like* object in Fig. 1b belongs to the group of suspended loads. Motivated by an extended workspace, mechanisms with single [8] and double [51] cable-suspensions were designed and controlled via parametric excitation to perform point to point motion and trajectory tracking. An impressive example of workspace extension is presented in [9], where a quadrotor injects energy into its suspended load such that it can pass through a narrow opening, which would be impossible with the load hanging down. The pendulum-like object in Fig. 1b is similar to the suspended loads of [50] and [51]. However, the former work focuses on oscillation damping and the latter uses one centralized controller.

In contrast to pendulum-like objects, *rigid* objects tightly couple the robot and the human motion. Thus, during human–robot cooperative swinging of rigid objects as illustrated in Fig. 1c, the robot needs to move “human-like” to allow for comfort on the human side. On this account, we conducted a pilot study on human–human rigid object swinging reported in [15]. The observed motion and frequency characteristics suggest that the human arm can be approximated as a torque-actuated simple pendulum with pivot point in front of the human shoulder. This result is in line with the conclusion drawn in [22] that the preferred frequency of a swinging lower human arm is dictated by the physical properties of the limb rather than the central nervous system.

Manipulation of *flexible* and deformable objects is a challenging research topic also at slow velocities. While the finite elements method aims at exact modeling [28], the pseudo-rigid object method offers an efficient tool to estimate deformation and natural frequency [49].

Here, instead of aiming for an accurate model, we achieve stable oscillations of unknown flexible objects by making use of the fact that the desired oscillation is *simple pendulum-like*. Simple pendulum approximations have been successfully used to model and control complex mechanisms, e.g., for brachiating [36] or dancing [46]. The swing-up and stabilization of simple pendulums in their unstable equilibrium point is commonly used as benchmark for linear and nonlinear control techniques [1, 18]. Instead of a full swing-up to the inverted pendulum configuration, our goal is to reach a periodic motion of desired energy content. Based on virtual holonomic constraints, [19] and achieve desired periodic motions. Above controllers rely on thorough system knowledge, whereas our final goal is the manipulation of *unknown flexible objects*.

### Adaptive Control for Periodic Motions and Leader–Follower Behavior

The cooperative sawing task in [38] is achieved via learning of individual dynamic movement primitives for motion and stiffness control with a human tutor in the loop. Frequency and phase are extracted online by adaptive frequency oscillators [39]. The applicability of learning methods as learning from demonstration [4] or reinforcement learning [16] to nonlinear dynamics is frequently evaluated based on inverted pendulum tasks. Reinforcement learning often suffers from the need of long interactions with the real system and from a high number of tuning parameters [35, 37]. Only recently, Deisenroth *et al.* showed how Gaussian processes allow for faster autonomous reinforcement learning with few parameters in [10]. Neural networks constitute another effective tool to control nonlinear systems, which have also been applied to adaptive leader–follower consensus control in, e.g., [47].

In this work, we apply model knowledge of the swinging task to design adaptive leader/follower controllers for swinging of unknown flexible objects, without the need of a learning phase. Identification of the underlying fundamental dynamics allows us to design leader and follower controllers which only require few parameters of distinct physical meaning.

## Overview of the Fundamental Dynamics-Based Approach

**Fig. 2:**
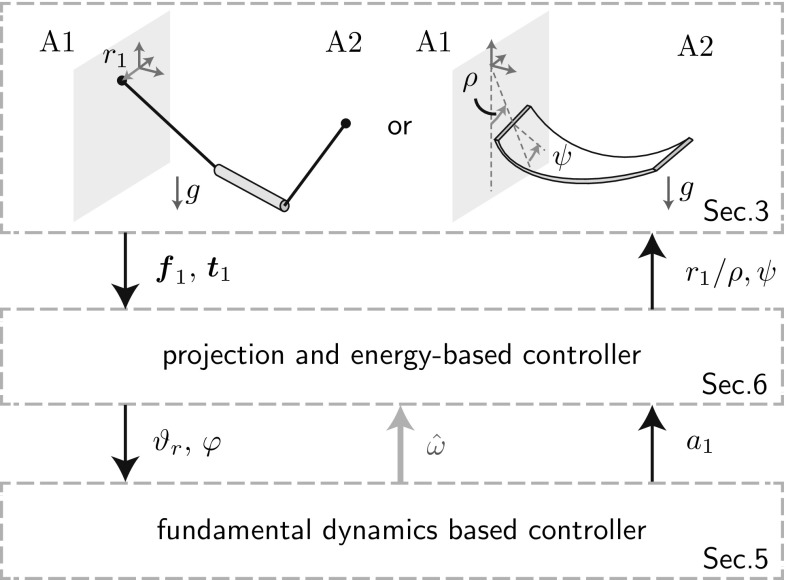
Implementation overview block diagram. Based on measured force $\varvec{f}_1$ and torque $\varvec{t}_1$, the complex afa-system and t-pendulum are projected onto their simple pendulum variants. From the extracted FD states $\varphi$ and $\vartheta _r$ the natural frequency is estimated ${\hat{\omega }}$ and leader or follower behavior is realized $a_1$. Energy-based controllers convert the amplitude factor $a_1$ into desired end effector motion defined by $r_1$ or $\rho$ and $\psi$

This section highlights the main ideas of the proposed approach and structures the article along Figs. 1 and 2. Individual variables will be introduced in subsequent sections and important variables are listed in Table 1.

In this work, we achieve cooperative energy injection into unknown flexible objects based on an understanding of the underlying desired *fundamental dynamics* (FD). Figure 1 illustrates the approximation steps taken that lead from human–robot flexible object swinging (a) to the FD (h). Pendulum-like objects (b) constitute the extreme end on the scale of flexible objects (a) with respect to the coupling strength between the agents. The especially weak coupling allows us to isolate the object from the agents’ end effectors and represent the agent’s influence by acceleration inputs. In the following, we refer to the isolated pendulum-like object (d) as *t-pendulum* due to its trapezoidal shape. In order to achieve our final goal of flexible object swinging, we consolidate our insights on pendulum and rigid object swinging (see step 2 in Fig. 1). We exploit the result that human arms behave as simple pendulums during rigid object swinging [15] and approximate the human arms by simple pendulums actuated via torque at the shoulder joints. We abbreviate the resultant “arm—flexible object—arm” system (e) as *afa-system*.

We do not try to extract accurate dynamical models, but make use of the fact that the desired oscillations are *simple pendulum-like*. The desired oscillations of the t-pendulum and the afa-system are then represented by cart-actuated (f) and torque-actuated (g) simple pendulums, respectively. We extract linear FD (h) which describes the phase and energy dynamics of the simple pendulum approximations controlled by a variant of the swing-up controller of Yoshida [48]. The FD allows for online frequency estimation (i), controlled energy injection and effort sharing among the agents (j).

**Table 1:** Important variables and abbreviations

FD	Fundamental dynamics
$\varvec{f}_i$ / $\varvec{t}_i$	Force/torque applied by agent *i*
$r_i, \dot{{r}}_i, \ddot{{r}}_i$	Position, velocity, acceleration of agent *i* in *x*-direction with respect to its initial position
$t_\mathrm {s}$	Torque applied at shoulder of virtual arm
$\theta$ / $\psi$	Desired/undesired oscillation DoF
$\rho$	Virtual arm deflection angle
$\vartheta$	Oscillation DoF of abstract simple pendulums
$\varphi$	Phase angle
*E*, $E_j$	Energy, energy of oscillation *j*
$j_E$	Amplitude of oscillation *j* (energy equivalent)
$\vartheta _r$	Phase space radius (approx. energy equivalent)
$a_i$	Amplitude factor of agent *i*
$\omega$	Natural frequency
$\omega _{0}$ / $\omega _\mathrm {g}$	Small angle/geometric mean approximation
$\varGamma _i$	Relative energy contribution of agent *i*
$(\cdot )_i$	Agent A*i*
$(\cdot )_{\mathcal {F}}$, $(\cdot )_{\mathcal {L}}$	Follower, leader agent
$(\cdot )_\mathrm {o}$ / $(\cdot )_\mathrm {a}$	Parameters of object/virtual arm
$(\cdot )_\mathrm {ref}$	Reference dynamics
$(\cdot )^*$	Projection of $(\cdot )$ onto *xy*-plane
$\hat{(\cdot )}$ / $(\cdot )^\mathrm {d}$	Estimate/ desired value of $(\cdot )$

The block diagram in Fig. 2 visualizes the implementation with input and output variables. The blocks will be detailed in the respective sections as indicated in Figs. 1 and 2. We would like to emphasize here that the proposed robot controllers generate desired end effector motion solely based on force and torque measurements at the robot’s interaction point.^1^

The remainder of the article is structured as follows. In Sect. 3 we give the problem formulation. This is followed by the FD derivations in Sect. 4, on which basis the adaptive leader and follower controllers are introduced and analyzed in Sect. 5. In Sect. 6, we apply the FD-based controllers to the two-agent t-pendulum and afa-system. We evaluate our controllers in simulation and experiments in Sects. 7 and 8, respectively. In Sect. 9, we discuss design choices, limitations and possible extensions of the presented control approach. Section 10 concludes the article.

## Problem Formulation for Cooperative Object Swinging

In this section, we introduce relevant variables and parameters of the t-pendulum and afa-system of Fig. 1d, e. Thereafter, we formally state our problem. Note that we drop the explicit notation of time dependency of the system variables where clear from the context.

**Fig. 3:**
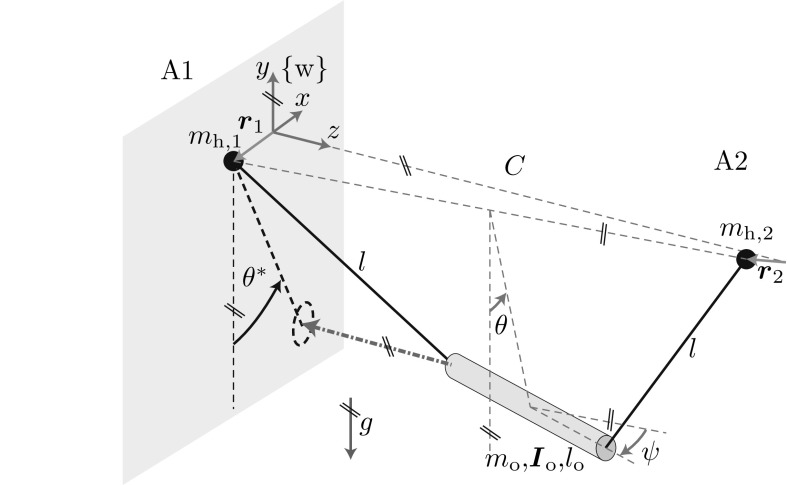
The t-pendulum (adapted from [13]): cylindrical object of mass $m_\mathrm {o}$, length $l_\mathrm {o}$ and moment of inertia $\varvec{I}_\mathrm {o}$ under the influence of gravity *g* attached via massless ropes of length *l* to two handles of mass $m_{\mathrm {h},i}$ located at $\varvec{r}_{i}$ with $i=1,2$. The location $\varvec{r}_{1}$ is defined with respect to the world fixed coordinate system $\{\mathrm {w}\}$. The location $\varvec{r}_{2}$ is defined with respect to the fixed point $^\mathrm {w} \varvec{p}=\left[ 0,\; 0 ,\; C\right] ^\top$ in $\{\mathrm {w}\}$, where *C* is the initial distance between the two agents. Pairs of *parallel lines* at the same angle indicate parallelity

### The t-Pendulum

Figure 3 shows the t-pendulum. Without loss of generality, we assume that agent A1 $=$ R is the robot who cooperates with a human A2 $=$ H. The t-pendulum has 10 degrees of freedom (DoFs), if we assume point-mass handles: the 3D positions of the two handles $\varvec{r}_{1}$ and $\varvec{r}_{2}$ representing the interaction points of the two agents A1 and A2 and 4 oscillation DoFs. The oscillation DoF $\theta$ describes the desired oscillation and is defined as the angle between the *y*-axis and the line connecting the center between the two agents and the center of mass of the pendulum object. The oscillation DoF $\psi$ describes oscillations of the object around the *y*-axis and is the major undesired oscillation DoF. Experiments showed that oscillations around the object centerline and around the horizontal axis perpendicular to the connection line between the interaction partners^2^ play a minor role and are therefore neglected in the following.

The agents influence the t-pendulum by means of handle accelerations $\ddot{\varvec{r}}_{1}$ and $\ddot{\varvec{r}}_{2}$. Although we assume cooperating agents, the only controllable quantity of agent $\mathrm {A}1$ is its own acceleration $\ddot{\varvec{r}}_{1}$. The acceleration $\ddot{\varvec{r}}_{2}$ of agent $\mathrm {A}2$ acts as a disturbance as it cannot be directly influenced by agent $\mathrm {A}1$. We limit the motion of agent $\mathrm {A}1$ to the *x*-direction for simplicity, which yields the one dimensional input ${u}_\mathrm {1}=\ddot{{r}}_{1}$. Experiments showed that 1D motion is sufficient and does not disturb a human interaction partner in comfortable 3D motion, because the pendulum-like object only loosely couples the two agents. The forces applied at the own handle are the only measurable quantity of agent A1, i.e. measurable output $\varvec{y}_{1}=\varvec{f}_1$.

**Fig. 4:**
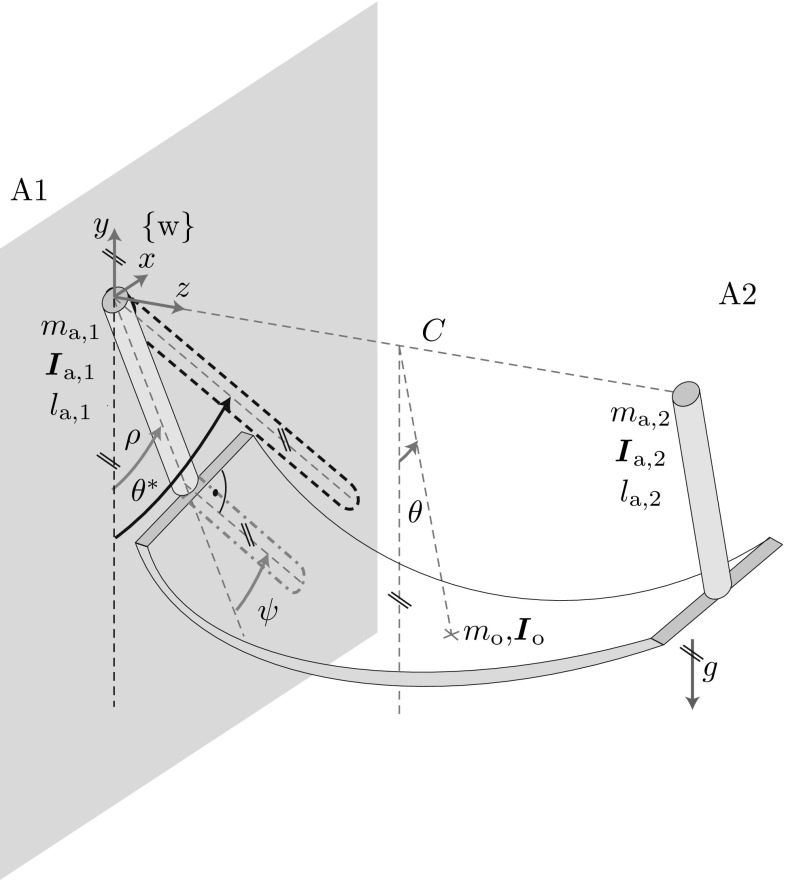
The afa-system: two cylindrical arms connected at their wrist joints through a flexible object of mass $m_{\mathrm {o}}$ and deformation dependent moment of inertia $\varvec{I}_{\mathrm {o}}$ under the influence of gravity *g*. The two cylindrical arms are of mass $m_{\mathrm {a},i}$, moment of inertia $\varvec{I}_{\mathrm {a},i}$ and length $l_{\mathrm {a},i}$ with $i=1,2$ and have their pivot point at the origin of the world fixed coordinate system $\{\mathrm {w}\}$ and at $^\mathrm {w} \varvec{p}=\left[ 0,\; 0 ,\;C\right] ^\top$ in $\{\mathrm {w}\}$, respectively. Pairs of *parallel lines* at the same angle indicate parallelity

### The afa-System

Figure 4 shows the afa-system. The cylindrical arms are actuated by shoulder torque around the *z*-axis $t_{s,1}$ and $t_{s,2}$. For simplicity, we limit the arm of agent $\mathrm {A}1$ to rotations in the *xy*-plane. Note that we use the same approximations for the side of agent $\mathrm {A}2$ for ease of illustration, although a human interaction partner can move freely. The angle between the negative *y*-axis and the arm of agent $\mathrm {A}1$ is the oscillation DoF $\rho$. The angle $\psi$ describes the wrist orientation with respect to the arm in the *xy*-plane (see right angle marking in Fig. 4). Thus, position and orientation of the interaction point of A1 are defined by the angles $\rho$ and $\psi$. We regard excessive and unsynchronized $\psi$-oscillations as undesired. The wrist joint is subject to damping $d_\psi$ and stiffness $k_\psi$. The desired oscillation DoF $\theta$ is defined as the angle between the *y*-axis and the line connecting the center between the two agents and the center of mass of the undeformed flexible object (indicated by a cross in Fig. 4). The input to the afa-system from the perspective of agent $\mathrm {A}1$ is its shoulder torque ${u}_{1}=t_{\mathrm {s},1}$. Agent $\mathrm {A}1$ receives force and torque signals at its wrist: measurable output $\varvec{y}_{1}=\left[ \varvec{f}_{1}^\top \;\varvec{t}_{1}^\top \right] ^\top$.

### Problem Statement

Our goal is to excite the desired oscillation $\theta$ to reach a periodic orbit of desired energy level $E^\mathrm {d}_\theta$ and zero undesired oscillation $E^\mathrm {d}_\psi =0$. The desired energy $E^\mathrm {d}_\theta$ is then equivalent to a desired maximum deflection angle $\theta ^\mathrm {d}_E$ or a desired height $h^\mathrm {d}_E$, at which the object could potentially be released. We define the energy equivalent $\varTheta _E$ for a general oscillation $\varTheta$:

#### Definition 1

*The energy equivalent* $\varTheta _E \in \left[ 0,\; \pi \right]$ *is a continuous quantity which is equal to the maximum deflection angle the* $\varTheta$-*oscillation would reach at its turning points* (${\dot{\varTheta }}=0$) *in case* $E_\varTheta ={const.}$

For the rest of the article, we interchangeably use $E_\theta$, $E_\psi$ and $\theta _E$, $\psi _E$ according to Definition 1 with $\varTheta =\theta ,\psi$ to refer to the energies contained in the $\theta$- and $\psi$-oscillations, respectively.

We differentiate between leader and follower agents. For a *leader* $\mathrm {A1}={\mathcal {L}}$ the control law ${u}_{\mathcal {L}}$ is a function of the measurable output $\varvec{y}_{\mathcal {L}}$ and the desired energy $\theta ^\mathrm {d}_E$. We formulate the control goal as follows1Hence, the energy of the $\theta$-oscillation should follow first-order reference dynamics $\theta _{E\mathrm {ref}}$ within bounds $\epsilon _\theta$. The reference dynamics are of inverse time constant $K_\mathrm {d}$ and converge to the desired energy $\theta ^\mathrm {d}_E$. Furthermore, the energy contained in the $\psi$-oscillation should stay within $\pm \epsilon _\psi$ after the settling time $T_\mathrm {s}$. We only consider desired energy levels of $\theta ^\mathrm {d}_E<\pi /2$ to avoid undesired phenomena as, e.g., slack suspension ropes in case of the pendulum-like object.

A *follower* $\mathrm {A1}={\mathcal {F}}$ does not know the desired energy level $\theta ^\mathrm {d}_E$. We define a desired relative energy contribution for the follower $\varGamma _{\mathcal {F}}^\mathrm {d}\in \left[ 0,\; 1 \right)$ based on the integrals over the energy flows of the leader $\dot{\theta }_{E,{\mathcal {L}}}$ and the follower $\dot{\theta }_{E,{\mathcal {F}}}$

2 $$\begin{aligned} {\varGamma _{\mathcal {F}}}=\frac{\int _0^{{T_\mathrm {s}}}{\dot{\theta }_{E,{\mathcal {F}}} \mathrm {d}\tau }}{\int _0^{{T_\mathrm {s}}}{({\dot{\theta }}_{E,{\mathcal {F}}} + {\dot{\theta }}_{E,{\mathcal {L}}}) \mathrm {d}\tau }}. \end{aligned}$$

Our goal is to split the energy effort among the leader and the follower such that the follower has contributed the fraction $\varGamma _{\mathcal {F}}^\mathrm {d}$ within bounds $\epsilon _{\mathcal {F}}$ at the settling time $T_\mathrm {s}$. To this end, we formulate the follower control goal as3The energy of the undesired oscillation $\psi _E$ should be kept within $\pm \epsilon _\psi$.

## Fundamental Dynamics

In this section, we introduce the abstract cart-pendulum and abstract torque-pendulum as approximations for the desired system oscillations of the t-pendulum and the afa-system (see Fig. 1d–g). This is followed by an introduction of the energy-based controller. Finally, we present the fundamental dynamics (FD) of the cart-pendulum and abstract torque-pendulum, which result from a state transformation, insertion of the energy-based controller and subsequent approximations.

### The Abstract Cart-Pendulum

For the ideal case of $\psi _E=0$ and agents that move along the *x*-direction in synchrony ${r}_{1}={r}_{2}$, the desired deflection angle $\theta$ is equal to the projected deflection angle $\theta ^*$ (projection indicated by the dashed arrow in Fig. 3). This observation motivates us to approximate the desired system behavior of the pendulum-like object as a cart-pendulum with two-sided actuation (see Fig. 1f)

4 $$\begin{aligned} {\dot{\varvec{x}}}_\mathrm {c}= \begin{bmatrix} {\dot{\vartheta }}\\ -\omega _{0}^2\sin \vartheta \end{bmatrix} +\begin{bmatrix} 0\\ -\frac{1}{g}\omega _{0}^2\cos \vartheta \end{bmatrix} \frac{{\ddot{r}}_{{1}}+{\ddot{r}}_{{2}}}{2}, \end{aligned}$$

with reduced state ${\varvec{x}}_\mathrm {c}=[ \vartheta ,\; {\dot{\vartheta }} ]^\top$ consisting of deflection angle $\vartheta$ and angular velocity ${\dot{\vartheta }}$ and the small angle approximation of the natural frequency $\omega _{0}$. We use the variables $\vartheta$ for the deflection angle of the abstract simple pendulum variants in contrast to the actual deflection angle $\theta$ of the complex objects. On the desired periodic orbit we have $\theta =\theta ^*=\vartheta$. The small angle approximation of the natural frequency $\omega _{0}=\frac{m_\vartheta c_\vartheta g}{I_\vartheta }$ depends on gravity *g* and abstract pendulum parameters: mass $m_\vartheta$, distance between pivot point and the center of mass $c_\vartheta$ and the resultant moment of inertia around the pendulum pivot point $I_\vartheta$. The parameters $m_\vartheta$ and $I_\vartheta$ represent one side of the t-pendulum, i.e. half of the mass and moment of inertia of the pendulum mass. By dividing the input accelerations by 2 in (4), we consider the complete mass and moment of inertia of the t-pendulum. We call this pendulum *abstract cart-pendulum*, where *cart* refers to the actuation through horizontal acceleration. The term *abstract* emphasizes the simplification we make by approximating the agents’ influences as summed accelerations and neglecting $\psi _E \ne 0$.

### The Abstract Torque-Pendulum

The afa-system simplifies to the two-link pendubot [43] with oscillation DoFs $\rho$ and $\psi$, when being projected into the *xy*-plane of agent A1 (see gray dash-dotted link in Fig. 4). For $\psi ^\mathrm {d}_E=0$, the pendubot further reduces to a single link pendulum actuated through shoulder torques of agents A1 and $\mathrm {A}2$ (see Fig. 1g)

5 $$\begin{aligned} {\dot{\varvec{x}}}_\mathrm {c}= \begin{bmatrix} {\dot{\vartheta }}\\omega_{0}^2\sin \vartheta \end{bmatrix} +\begin{bmatrix} 0\\ \frac{1}{I_\vartheta }\end{bmatrix} \frac{t_{\mathrm {s},1}+t_{\mathrm {s},2}}{2}. \end{aligned}$$

We call this pendulum *abstract torque-pendulum*. As for the abstract cart-pendulum, the parameter $I_\vartheta$ represents the moment of inertia of one side of the afa-system. Similar to the t-pendulum, we define a projected deflection angle $\theta ^*=\rho +\psi$ (see Fig. 4). On the desired periodic orbit we have $\theta =\theta ^*=\vartheta$.

### Energy-Based Control for Simple Pendulums

Here, we recapitulate important simple pendulum fundamentals and introduce the energy-based controller to be applied to the abstract simple pendulums. For the following derivations, we assume zero handle velocity for the cart-pendulum ${\dot{r}}_{1} = {\dot{r}}_{2} = 0$, which is the case for the torque-pendulum by construction. The energy contained in both abstract pendulums is then

6 $$\begin{aligned} E_\vartheta = {I_\vartheta {\dot{\vartheta }}^2 + 2 m_\vartheta g c_\vartheta \left( 1-\cos \vartheta \right) }. \end{aligned}$$

According to Definition 1, the energy equivalent $\vartheta _E$ is equal to the maximum deflection angle $\vartheta$ reached at the turning points for angular velocity $\dot{\vartheta }=0$

7 $$\begin{aligned} E_\vartheta = { 2 m_\vartheta } g c_\vartheta \left( 1-\cos \vartheta _{E}\right) . \end{aligned}$$

Setting (6) equal to (7), we can express $\vartheta _E$ in terms of the state $\varvec{x}_\mathrm {c}=\left[ \vartheta , \; \dot{\vartheta } \right] ^\top$

8 $$\begin{aligned} \vartheta _E=\arccos \left( \cos \vartheta -\frac{1}{2 \omega _{0}^2}{\dot{\vartheta }}^2\right) , \end{aligned}$$

with $\vartheta _E \in \left[ 0,\; \pi \right]$. In contrast to the energy $E_\vartheta$, which also depends on mass and moment of inertia of the object, the amplitude $\vartheta _E$ only depends on the small angle approximation of the natural frequency $\omega _{0}$. Therefore, we will use $\vartheta _E$ as the preferred energy measure in the following.

**Fig. 5:**
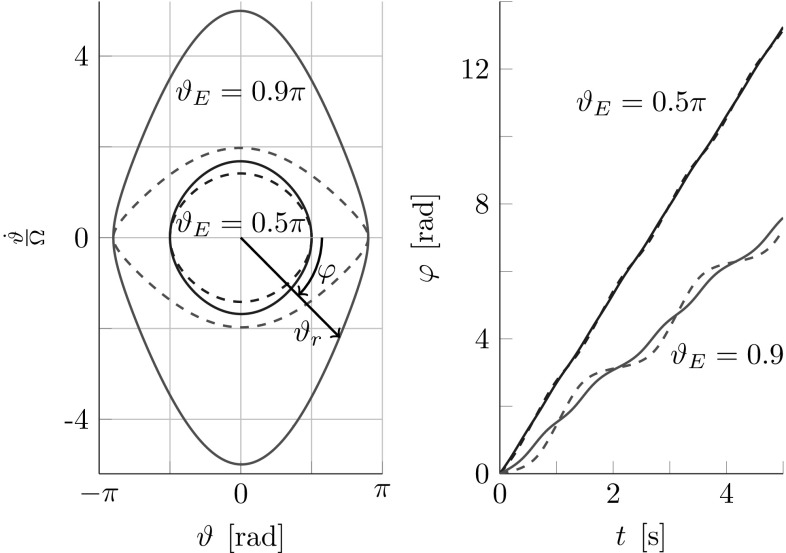
Phase portrait (*left*) and phase angle $\varphi$ over time (*right*) at constant energy levels $\vartheta _E=0.5\pi$ (*blue*) and $\vartheta _E=0.9\pi$ (*red*) of a lossless simple pendulum. Normalization with $\varOmega =\omega _\mathrm {g}$ marked via *solid lines* and $\varOmega =\omega _{0}$ via *dashed lines*. For energies up to $\vartheta _E=0.5\pi$ and a normalization with $\varOmega =\omega _\mathrm {g}$, the phase space is approximately a circle with radius $\vartheta _r \approx \vartheta _E$ and the phase angle $\varphi$ rises approximately linear over time. Figure adapted from [14]

Simple pendulums constitute nonlinear systems with an energy dependent natural frequency $\omega (\vartheta _E)$. No analytic solution exists for $\omega$, but it can be obtained numerically by $\omega =\omega _0 M\left\{ 1,\cos \frac{\vartheta _{E}}{2}\right\}$ with the arithmetic-geometric mean $M\left\{ x,y \right\}$ [6]. Already the first iteration of $M\left\{ 1,\cos \frac{\vartheta _{E}}{2}\right\}$ yields good estimates for $\omega$

9 $$\begin{aligned} \omega \approx \left\{ \begin{array}{cl} \omega _\mathrm {a}=\omega _0\frac{1+\cos \frac{\vartheta _{E}}{2}}{2}&{}\\ \omega _\mathrm {g}=\omega _0\sqrt{\cos \frac{\vartheta _{E}}{2}}&{}, \end{array}\right. \end{aligned}$$

with relative error 0.748 % for the arithmetic mean approximation $\omega _\mathrm {a}$ and 0.746 % for the geometric mean approximation $\omega _\mathrm {g}$ at $\vartheta _E=\frac{\pi }{2}$ with respect to the sixth iteration of $M\left\{ 1,\cos \frac{\vartheta _{E}}{2}\right\}$. In the following, we make use of the geometric mean approximation $\omega _\mathrm {g}$ within derivations and as ground truth for comparison to the estimate ${\hat{\omega }}$ in simulations and experiments.

The pendulum nonlinearities are visualized in phase portraits on the left side of Fig. 5 for two constant energy levels $\vartheta _E=0.5\pi$ and $\vartheta _E=0.9\pi$. The inscribed phase angle $\varphi$ is

10 $$\begin{aligned} \varphi = {{\mathrm{atan2}}} \left( -\frac{{\dot{\vartheta }}}{\varOmega } , \vartheta \right) , \end{aligned}$$

with normalization factor $\varOmega$. The right side of Fig. 5 displays the phase angle $\varphi$ over time. The normalization factor $\varOmega$ is used to partly compensate for the pendulum nonlinearities, with the result of an almost circular phase portrait and an approximately linearly rising phase angle

11 $$\begin{aligned} \varphi (t) \approx \omega t + \varphi (t=0). \end{aligned}$$

Figure 5 shows that normalization with the more accurate geometric mean approximation of the natural frequency $\varOmega =\omega _\mathrm {g}$ allows for a better compensation of the pendulum nonlinearities than a normalization with the small angle approximation $\varOmega =\omega _{0}$.

The main idea of the energy control for the abstract cart-pendulum is captured in the control law [48]

12 $$\begin{aligned} {\ddot{r}}_{i} = a_{i} \; \omega ^2 \sin \varphi , \end{aligned}$$

where the amplitude factor $a_{i}$ regulates the sign and amount of energy flow contributed by agent A*i* to the abstract cart-pendulum, with $i=1,2$. A well-timed energy injection is achieved through multiplication with $\sin \varphi$, which according to (11) excites the pendulum at its natural frequency. For the abstract torque-pendulum we choose a similar control law with

13 $$\begin{aligned} t_{\mathrm {s},i} = - a_{i} \sin \varphi . \end{aligned}$$

### Cartesian to Polar State Transformation

The abstract cart- and torque-pendulum dynamics in (4) and (5) are nonlinear with respect to the states $\varvec{x}_\mathrm {c}=[ \vartheta ,\; \dot{\vartheta } ]^\top$. The index $\mathrm {c}$ indicates that the angle $\vartheta$ and angular velocity $\dot{\vartheta }$ represent the cartesian coordinates in the phase space (see left side of Fig. 5). We expect the system energy $\vartheta _E$ to ideally be independent of the phase angle $\varphi$, which motivates a state transformation to $\varphi$ and $\vartheta _E$ for simple adaptive control design. Solving (10) for $\dot{\vartheta }$ and insertion into (8) yields

14 $$\begin{aligned} \cos \vartheta _E=\cos \vartheta -\frac{\varOmega ^2}{2 \omega _{0}^2}{\tan ^2(\varphi )} \; \vartheta ^2. \end{aligned}$$

However, there is no analytic solution for $\vartheta (\vartheta _E,\varphi )$ from (14). Therefore, we approximate the system energy $\vartheta _E$ through the phase space radius $\vartheta _r$

15 $$\begin{aligned} \vartheta _{r}:=\sqrt{\vartheta ^2+\left( \frac{{\dot{\vartheta }}}{\varOmega } \right) ^2}. \end{aligned}$$

From Fig. 5 we see that the phase space radius is equal to the energy $\vartheta _r = \vartheta _E$ at the turning points (${\dot{\vartheta }}=0$). For energies $\vartheta _E\le \frac{\pi }{2}$ and a normalization with $\varOmega \approx \omega$, the phase space is almost circular and thus $\vartheta _r \approx \vartheta _E$ also for ${\dot{\vartheta }}\ne 0$.

The phase angle $\varphi$ and the phase space radius $\vartheta _r$ span the polar state space $\varvec{x}_\mathrm {p}=\left[ \varphi , \; \vartheta _r \right] ^\top$, which we mark with the subscript $\mathrm {p}$. The cartesian states $\varvec{x}_\mathrm {c}$ written as a function of the polar states $\varvec{x}_\mathrm {p}$ are

16 $$\begin{aligned} \vartheta= & {} \vartheta _{r}\cos \varphi \\ {\dot{\vartheta }}= & {} -\vartheta _{r} \varOmega \sin \varphi .\nonumber \end{aligned}$$

### The Fundamental Dynamics

#### Theorem 1

The *FD* of the abstract cart- and torque-pendulums in (4) and (5) under application of the respective control laws (12) and (13) can be written in terms of the polar states $\varvec{x}_\mathrm {p}=\left[ \varphi ,\; \vartheta _r \right] ^\top$ as

17 $$\begin{aligned} \dot{\varvec{x}}_\mathrm {p} = \begin{bmatrix}{\dot{\varphi }} \\ {\dot{\vartheta }}_{r}\end{bmatrix} = \begin{bmatrix}\omega \\ 0\end{bmatrix} + \begin{bmatrix}0 \\ B \end{bmatrix} \frac{a_{1}+a_{2}}{2}, \end{aligned}$$

with system parameter

18 $$\begin{aligned} B=\left\{ \begin{array}{cl} B_{{\ddot{r}}} =\frac{1}{2g}\omega ^3 &{} \text {abstract cart-pendulum}, \\ B_{t} =\frac{1}{2\omega I_\vartheta }&{} \text {abstract torque-pendulum}, \end{array} \right. \end{aligned}$$

when neglecting higher harmonics, applying 3rd order Taylor approximations and making use of the geometric mean approximation of the natural frequency $\omega _\mathrm {g}$ in (9).

#### Proof

See “Appendix A”. $$\square $$

Thus, the phase $\varphi$ is approximately time-linear $\dot{\varphi } \approx \omega$ and the influence of the actuation *a* on the phase is small. The energy flow $\dot{\vartheta }_E \approx \dot{\vartheta }_r$ is approximately equal to the mean of the amplitude factors $a_{1}$ and $a_{2}$ times a system dependent factor *B*, and thus zero for no actuation $a_{1}=a_{2}=0$.

## FD-Based Adaptive Leader–Follower Structures

In this section, we use the fundamental dynamics (FD) to design adaptive controllers that render leader and follower behavior according to (1) and (3). For the abstract cart-pendulum FD, the natural frequency $\omega$ is the only unknown system parameter. For the abstract torque-pendulum, also an estimate of the moment of inertia ${\hat{I}}_\vartheta$ is required. Here, we first present the natural frequency estimation. In Sect. 6.3, we discuss how to obtain ${\hat{I}}_\vartheta$. The $\omega$-estimate is not only needed for the computation of the system parameter *B*, but also for the phase angle $\varphi$, required in the control laws (12) and (13). In a second step, we design the amplitude factor $a_{1}$ to render either leader or follower behavior.

### Estimation of Natural Frequency

Based on the phase FD ${\dot{\varphi }} = \omega$, we design simple estimation dynamics for the natural frequency estimate ${\hat{\omega }}$

19 $$\begin{aligned} \hat{\omega }= \frac{s}{1+T_\omega s} \varphi , \end{aligned}$$

which differentiates $\varphi$, while also applying a first-order low-pass filter with cut-off frequency $\frac{1}{T_\omega }$.

**Fig. 6:**
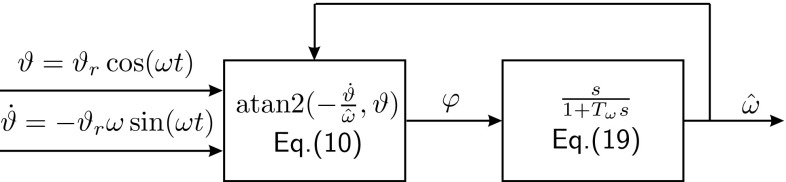
Block diagram of the $\omega$-estimation with normalization factor $\varOmega =\hat{\omega }$ used for the computation of phase angle $\varphi$

Figure 6 shows how the $\omega$-estimation is embedded into the controller. The feedback of the estimate ${\hat{\omega }}$ for the computation of phase angle $\varphi$ requires a stability analysis.

#### Proposition 1

The natural frequency estimate ${\hat{\omega }}$ converges to the true natural frequency $\omega$ when estimated according to Fig. 6 with

20 $$\begin{aligned} {T_\omega> \max \left( \frac{1}{2 {\hat{\omega }}(t=0)}, \frac{1}{2 \omega } \right) \; \text {and} \quad {\hat{\omega }}(t=0)>0,} \end{aligned}$$

and if the system behaves according to the FD with constant natural frequency $\omega$ ($\omega$ changes only slowly w.r.t. the ${\hat{\omega }}$-dynamics in (19)).

#### Proof

See “Appendix B”. $$\square $$

Condition (20) indicates that the adaptation of ${\hat{\omega }}$ cannot be performed arbitrarily fast.

### Amplitude Factor Based Leader/Follower Design

In the following, we design the amplitude factors for leader agents $a_{\mathcal {L}}$ and follower agents $a_{\mathcal {F}}$.

#### Leader ${\mathcal {L}}$

##### Proposition 2

For two *leader* agents $\mathrm {A1}=\mathrm {A2}={\mathcal {L}}$ applying amplitude factors

21 $$\begin{aligned} a_i=k_i(\theta ^\mathrm {d}_E-\vartheta _r) \text { with } k_i=\frac{2 \varGamma ^\mathrm {d}_i K_\mathrm {d}}{{B}}, \end{aligned}$$

where $i=1,2$, $\varGamma ^\mathrm {d}_1+\varGamma ^\mathrm {d}_2=1$, and $\vartheta _{r}(t=0)=\theta _{E\mathrm {ref}}(t=0)$, the energy $\theta _r$ of the FD in (17) converges to the desired energy $\theta _E^\mathrm {d}$ and tracks the desired reference dynamics in (1)

22 $$\begin{aligned} {\dot{\theta }}_{E\mathrm {ref}}= & {} K_{\mathrm {d}}\left( \theta _{E}^{\mathrm {d}}-\theta _{E\mathrm {ref}}\right) . \end{aligned}$$

Furthermore, each leader agent contributes with the desired relative energy contribution $\varGamma _i=\varGamma _i^\mathrm {d}$ defined in (2).

##### Proof

Differentiation with respect to time of the Lyapunov function

23 $$\begin{aligned} V= \frac{1}{2}\left( \theta _E^\mathrm {d}-\vartheta _r\right) ^2 \end{aligned}$$

and insertion of the FD (17) with (21) yields

24 $$\begin{aligned} {\dot{V}}= & {} -\frac{B}{2} (k_1+k_2)(\theta _E^\mathrm {d}-\vartheta _r)^2. \end{aligned}$$

Thus, as long as $\vartheta _{r}\ne \theta _{E}^\mathrm {d}$ and for $k_1+k_2,B>0$ the Lyapunov function has a strictly negative time derivative ${\dot{V}} <0$ and, thus, the desired energy level $\vartheta _{r}=\theta _{E}^\mathrm {d}$ is an asymptotically stable fixpoint.

Insertion of (21) into the FD in (17) yields

25 $$\begin{aligned} {\dot{\vartheta }}_{r}= K_{\mathrm {d}}\left( \theta _{E}^{\mathrm {d}}-\vartheta _{r}\right) . \end{aligned}$$

Comparison of (25) and (22) shows that the reference dynamics are tracked $\vartheta _{r}(t) =\theta _{E\mathrm {ref}}(t)$ for equal initial values $\vartheta _{r}(t=0)=\theta _{E\mathrm {ref}}(t=0)$. The energy contributed by one agent *i* according to the FD in (17) is $\dot{\vartheta }_{r,i}=\frac{B}{2} a_i$. Insertion of (21) yields $\dot{\vartheta }_{r,i}= \varGamma _i^\mathrm {d}K_{\mathrm {d}}\left( \theta _{E}^{\mathrm {d}}-\vartheta _{r}\right)$. With (25), the relative energy contribution of agent *i* according to (2) results in $\varGamma _i= \frac{\int _0^{T_\mathrm {s}} \dot{\vartheta }_{r,i} \mathrm {d}\tau }{\int _0^{T_\mathrm {s}} \dot{\vartheta }_{r} \mathrm {d}\tau }= \varGamma _i^\mathrm {d}$. $\square$

#### Follower ${\mathcal {F}}$

##### Proposition 3

A *follower* agent $\mathrm {A1}={\mathcal {F}}$ applying an amplitude factor

26 $$\begin{aligned} a_{\mathcal {F}}=k_{\mathcal {F}}\hat{\dot{\vartheta }}_r \text { with } k_{\mathcal {F}}=\frac{2}{B}\varGamma ^\mathrm {d}_{\mathcal {F}}, \end{aligned}$$

with $\varGamma ^\mathrm {d}_{\mathcal {F}} \in \left[ 0, 1 \right)$ and a correct estimate of the total energy flow $\hat{\dot{\vartheta }}_r={\dot{\vartheta }}_r$, contributes the desired fraction $\varGamma _{\mathcal {F}} = \varGamma {^\mathrm {d}}_{\mathcal {F}}$ to the overall task effort.

##### Proof

Insertion of (26) into the energy flow of the follower $\dot{\vartheta }_{r,{\mathcal {F}}}=\frac{B}{2} a_{\mathcal {F}}$ according to the FD in (17) yields $\dot{\vartheta }_{r,{\mathcal {F}}}=\varGamma ^\mathrm {d}_{\mathcal {F}} \hat{\dot{\vartheta }}_r$ and $\varGamma _{\mathcal {F}} = \varGamma {^\mathrm {d}}_{\mathcal {F}}$ (see proof or Proposition 2). $\square$

We obtain the total energy flow estimate through filtered differentiation $\hat{\dot{\vartheta }}_r=G_\mathrm {hp}(T_{\mathcal {F}}) {{\vartheta }}_r$, where $G_\mathrm {hp}(T_{\mathcal {F}})$ is a first-order high-pass filter with time constant $T_{\mathcal {F}}$. Thus, the filtered energy flow estimate is not equal to the true value $\hat{\dot{\vartheta }}_r \ne {\dot{\vartheta }}_r$. The influence of this filtering will be investigated in the next section.

**Fig. 7:**
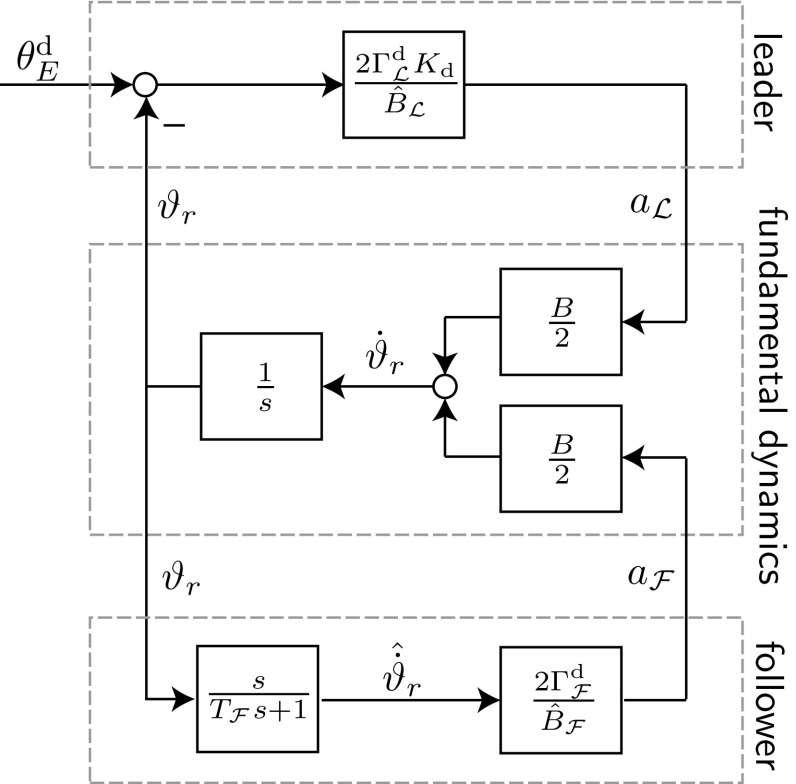
Block diagram showing the leader and follower controllers interacting with the linear fundamental energy dynamics. The leader tracks first-order reference dynamics with inverse time-constant $K_\mathrm {d}$ to control the energy $\vartheta _r$ to $\theta _E^\mathrm {d}$ with a desired relative energy contribution $\varGamma _{\mathcal {L}}^\mathrm {d}$. The follower achieves a desired relative energy contribution $\varGamma _{\mathcal {F}}^\mathrm {d}$ by imitating an estimate of the system energy flow $\hat{{\dot{\vartheta }}}_r$

### Analysis of Leader–Follower Structures

Here, we analyze stability, stationary transfer behavior and resultant follower contribution $\varGamma _{\mathcal {F}}$ for filtered energy flow estimates $\hat{\dot{\vartheta }}_r$ and estimation errors on the follower $B- {\hat{B}}_{\mathcal {F}}\ne 0$ and leader $B- {\hat{B}}_{\mathcal {L}}\ne 0$ side. Figure 7 shows a block diagram of the fundamental energy dynamics-based control structure for a leader and a follower controller. See “Appendix C” for details on the derivations of the transfer functions.

The reference transfer function $\vartheta _r(s)=G^{\mathrm {fi}}(s)\theta _E^\mathrm {d}(s)$, which describes the closed-loop behavior resulting from the interconnection depicted in Fig. 7, results in

27 $$\begin{aligned}&G^{\mathrm {fi}} = \nonumber \\&\quad \frac{\varGamma ^\mathrm {d}_{\mathcal {L}} K_\mathrm {d} \frac{B}{{\hat{B}}_{\mathcal {L}}} s+ \varGamma ^\mathrm {d}_{\mathcal {L}} K_\mathrm {d} \frac{B}{{\hat{B}}_{\mathcal {L}}} \frac{1}{T_{\mathcal {F}}}}{s^2 + \left( \frac{1}{T_{\mathcal {F}}}-\varGamma ^\mathrm {d}_{\mathcal {F}} \frac{B}{{\hat{B}}_{\mathcal {F}}}\frac{1}{T_{\mathcal {F}}} +\varGamma ^\mathrm {d}_{\mathcal {L}} K_\mathrm {d} \frac{B}{{\hat{B}}_{\mathcal {L}}} \right) s + \varGamma ^\mathrm {d}_{\mathcal {L}} K_\mathrm {d} \frac{B}{{\hat{B}}_{\mathcal {L}}} \frac{1}{T_{\mathcal {F}}}}.\nonumber \\ \end{aligned}$$

Thus, $\vartheta _r(t \rightarrow \infty )=\theta _E^\mathrm {d}$ and we have a stationary transfer behavior equal to one for a step of height $\theta _E^\mathrm {d}$ in the reference variable $\theta _E^\mathrm {d}(t)=\sigma (t)\theta _E^\mathrm {d}$. This result holds irrespective of estimation errors ${\hat{B}}_\mathcal {F/L} \ne B$. Asymptotic stability of the closed-loop system is ensured for $(\frac{1}{T_{\mathcal {F}}}-\varGamma ^\mathrm {d}_{\mathcal {F}} \frac{B}{{\hat{B}}_{\mathcal {F}}}\frac{1}{T_{\mathcal {F}}}+\varGamma ^\mathrm {d}_{\mathcal {L}} K_\mathrm {d} \frac{B}{{\hat{B}}_{\mathcal {L}}} )>0$. The stability constraint implies that ${\hat{B}}_{\mathcal {F}} > B$ is advantageous. This can be achieved by using a high initial value in the follower’s ${\hat{\omega }}$-estimation for the abstract cart-pendulum and a low initialization for the abstract torque-pendulum (see (18)). Factors such as estimation errors, a high desired follower contribution $\varGamma ^\mathrm {d}_{\mathcal {F}}$ and a small time constant $T_{\mathcal {F}}$ can potentially destabilize the closed-loop system.

**Fig. 8:**
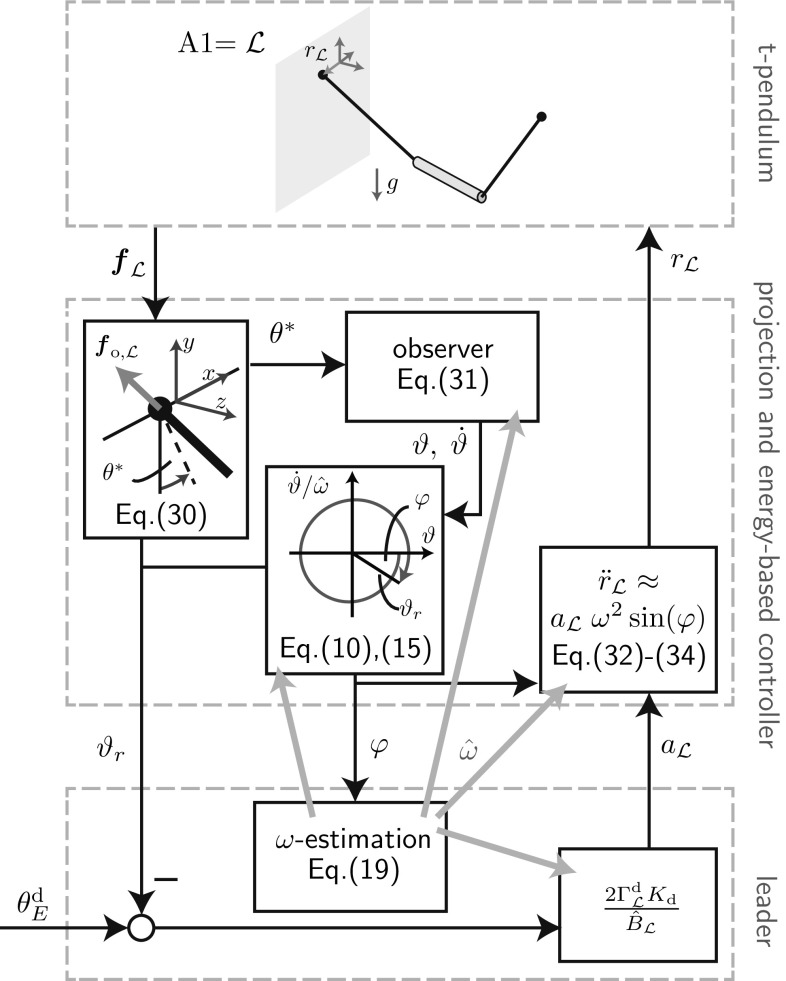
Block diagram of the FD-based leader applied to the t-pendulum

The follower transfer function $G^\mathrm {fi}_{\mathcal {F}}$ from desired energy level $\theta _E^\mathrm {d}$ to follower energy $\theta _{r{\mathcal {F}}}$ is

28 $$\begin{aligned}&G^\mathrm {fi}_{\mathcal {F}}=\nonumber \\&\quad \frac{\varGamma ^\mathrm {d}_{\mathcal {L}} K_\mathrm {d} \frac{B}{{\hat{B}}_{\mathcal {L}}} \varGamma ^\mathrm {d}_{\mathcal {F}} \frac{B}{{\hat{B}}_{\mathcal {F}}} \frac{1}{T_{\mathcal {F}}}}{s^2+\left( \frac{1}{T_{\mathcal {F}}} -\varGamma ^\mathrm {d}_{\mathcal {F}} \frac{B}{{\hat{B}}_{\mathcal {F}}}\frac{1}{T_{\mathcal {F}}} + \varGamma ^\mathrm {d}_{\mathcal {L}}K_\mathrm {d} \frac{B}{{\hat{B}}_{\mathcal {L}}}\right) s + \varGamma ^\mathrm {d}_{\mathcal {L}} K_\mathrm {d} \frac{B}{{\hat{B}}_{\mathcal {L}}} \frac{1}{T_{\mathcal {F}}}}. \end{aligned}$$

Application of the final value theorem to (28) yields $\vartheta _{r,{\mathcal {F}}}(t \rightarrow \infty )= \varGamma ^\mathrm {d}_{\mathcal {F}} \frac{B}{{\hat{B}}_{\mathcal {F}}} \theta _E^\mathrm {d}$. Consequently, $\varGamma _{\mathcal {F}}=\varGamma ^\mathrm {d}_{\mathcal {F}} \frac{B}{{\hat{B}}_{\mathcal {F}}}$ and the follower achieves its desired relative energy contribution for a correct estimate ${\hat{B}}_{\mathcal {F}}=B$.

**Fig. 9:**
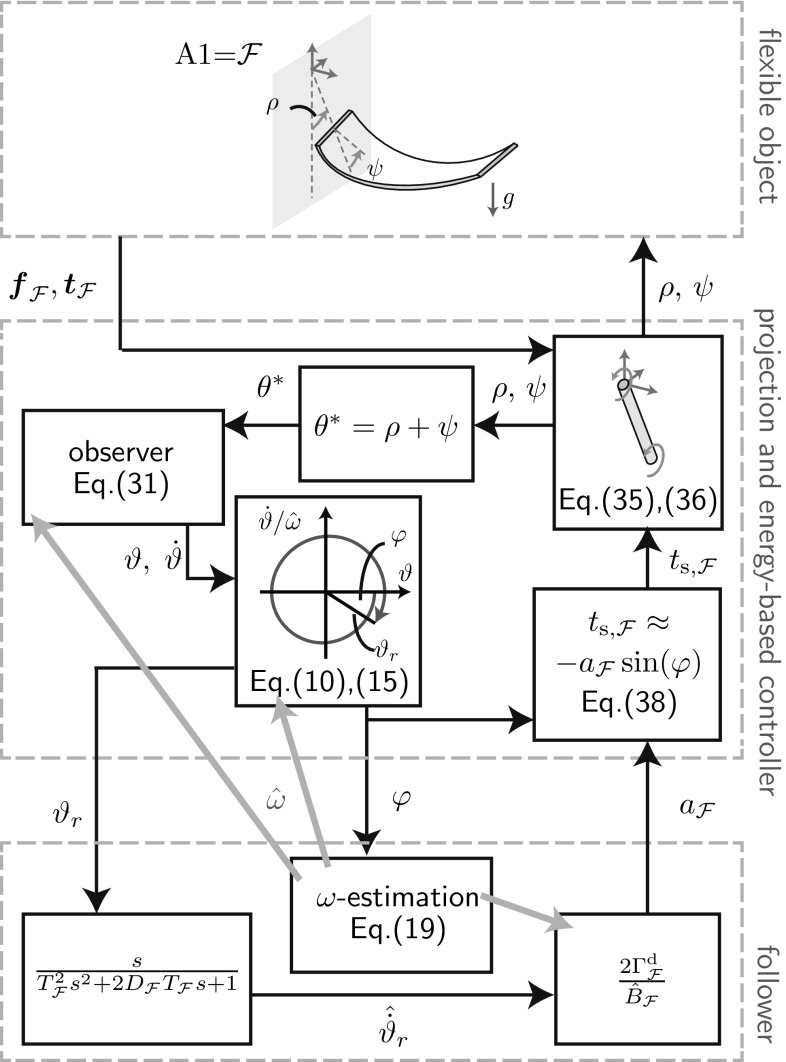
Block diagram of the FD-based follower applied to the afa-system

## Application to Two-Agent Object Manipulation

Here, we extend the fundamental dynamics (FD)-based adaptive controllers presented in the previous section to control the t-pendulum and the afa-system. Figures 8 and 9 show block diagrams of the controller implementation for the t-pendulum controlled by a leader agent and the afa-system controlled by a follower agent, respectively. *Follower* and *leader* controllers are invariant with respect to the object types. In Sect. 6.1, we discuss modifications of the fundamental dynamics-based controllers to cope with modeling errors. The *projection and energy-based controller* block differs between the t-pendulum and the afa-system and will be explained in detail in Sects. 6.2 and 6.3, respectively.

### FD-Based Controllers

The FD derivation is based on approximating the system energy $\vartheta _E$ by the phase space radius $\vartheta _r$ in Sect. 4.4. As visible in the phase space on the left side of Fig. 5, the phase space radius $\vartheta _r$ represents the system energy $\vartheta _E$ less accurately at higher energy levels. The effect is increased oscillations of $\vartheta _r$ for constant $\vartheta _E$. As a consequence, unsettled follower behavior is expected even when the leading partner is trying to keep the system energy at a constant level. Furthermore, the discrepancy between $\vartheta _r$ and $\vartheta _E$ degrades the leader’s reference dynamics tracking ability.

From $\vartheta$ and ${\dot{\vartheta }}$ we can estimate $\vartheta _E$ based on (8). To this end, we use the geometric mean relationship in (9) with current frequency estimate $\omega _g={\hat{\omega }}$ and solve it for the unknown small angle approximation ${\hat{\omega }}_0^2= {\hat{\omega }}^2 \left( \cos \left( {\hat{\vartheta }}_E/2 \right) \right) ^{-1}$. Insertion of ${\hat{\omega }}_0$ into (8) results in a quadratic equation which we solve for ${\hat{\vartheta }}_E$

29 $$\begin{aligned} {\hat{\vartheta }}_E = 2\arccos \left( -\frac{{\dot{\vartheta }}^2}{8 {\hat{\omega }}^2} + \frac{1}{4} \sqrt{\frac{{\dot{\vartheta }}^4}{4 {\hat{\omega }}^4} + 8 (\cos \vartheta +1) } \right) . \end{aligned}$$

The estimate ${\hat{\vartheta }}_E$ can now be used instead of $\vartheta _r$ within the leader and follower controllers.

Interestingly, the error caused by the phase space radius approximation has a greater influence on the abstract torque-pendulum than on the abstract cart-pendulum. Because $t_{\mathrm {s},1}$ in (13) and ${\dot{\vartheta }}$ reach their maxima for $\varphi =\pm \frac{\pi }{2}$, the torque-based actuation contributes maximum energy when the error between $\vartheta _r$ and $\vartheta _E$ has its maximum (see Fig. 5). In contrast, the acceleration-based actuation in (12) contributes most energy when the multiplication of velocity ${\dot{r}}_1$ and applied force in *x*-direction reach a maximum, where ${\dot{r}}_1$ has its maximum at $\varphi =0,\pi$. We will show the implications of above discussion and the usage of ${\hat{\vartheta }}_E$ based on simulations of the abstract simple pendulums in Sect. 7.

The realistic pendulum-like and flexible object do not exhibit perfect simple pendulum-like behavior. As we show with our experimental results in Sect. 8, such unmodeled dynamics have only little effect on the leader controller performance. In order to achieve calm follower behavior during constant energy phases, we use a second-order low-pass filter along with the differentiation of $\vartheta _r$ for the experiments instead of the first-order low-pass filter (compare Figs. 7, 9). Besides the extension by the $\omega$-estimation, the second-order filter for the follower is the only modification we apply to the FD-based controllers in Fig. 7 for the experiments. Because we are limited to relatively small energies for the afa-system where $\vartheta _r \approx \vartheta _E$, use of the more accurate estimate ${\hat{\vartheta }}_E$ is not needed.

At small energy levels, noise and offsets in the force and torque signals can lead to a phase angle $\varphi$ that does not monotonically increase over time. We circumvented problems with respect to the $\omega$-estimation by reinitializing ${\hat{\omega }}$ whenever $\vartheta _r$ decreased below a small threshold. No modifications were needed for the amplitude factor computation.

The computation of the FD parameter *B* in (18) requires a moment of inertia estimate ${\hat{I}}_\vartheta$. For the experiments, we computed ${\hat{I}}_\vartheta$ based on known parameters of the simple pendulum-like arm $I_{\vartheta \mathrm {a}}=I_{\mathrm {a}} + m_\mathrm {a} (\frac{l_\mathrm {a}}{2})^2$ and based on a point mass approximation of the flexible object ${\hat{I}}_{\vartheta \mathrm {o}}=\frac{m_\mathrm {o}}{2} (l_\mathrm {a}+{\hat{l}}^*_\mathrm {o})^2$. The part of the object mass carried by the robot $\frac{m_\mathrm {o}}{2}$ is measured with the force sensor. We furthermore assume that an estimate of the projected object length ${\hat{l}}^*_\mathrm {o}$ is available. Alternatively, the object moment of inertia could be estimated from force measurements during manipulation (e.g., [3, 27]).

### Projection and Energy-Based Controller for the t-Pendulum

#### Projection onto the Abstract Cart-Pendulum

The goal of what we call the *projection onto the abstract cart-pendulum*, is to extract the desired oscillation $\theta$ from the available force measurements $\varvec{f}_1$. The projection is performed in two steps. First, the projected deflection angle $\theta ^*$ is computed from $\varvec{f}_1$

30 $$\begin{aligned} \theta ^*=\arctan \left( \frac{-f_{\mathrm {o},1x}}{f_{\mathrm {o},1y}}\right) , \end{aligned}$$

with $\varvec{f}_{\mathrm {o},1}=\left[ {f}_{\mathrm {o},1x} ,\; {f}_{\mathrm {o},1y} ,\; {f}_{\mathrm {o},1z} \right] ^\top$ being the force exerted by agent A1 onto the pendulum-like object. We obtain $\varvec{f}_{\mathrm {o},1}$ from the measurable applied force $\varvec{f}_1$ through dynamic compensation of the force accelerating the handle mass $m_{\mathrm {h},1}$: $\varvec{f}_{\mathrm {o},1}=\varvec{f}_1-m_{\mathrm {h},1} \left[ {\ddot{r}}_1,\; -g,\; 0 \right] ^\top$.

The projected deflection angle $\theta ^*$ does not only contain the desired $\theta$-oscillation, but is superimposed by undesired oscillations, such as the $\psi$-oscillation in Fig. 3. In a second step, we apply a nonlinear observer to extract the states of the virtual abstract cart-pendulum

31 $$\begin{aligned} \dot{{\varvec{x} } }_\mathrm {c} = \begin{bmatrix} \dot{\vartheta } \\ -{\hat{\omega }}_0 \sin (\vartheta ) \end{bmatrix} + \varvec{l} (\theta ^* - y), \quad y= \begin{bmatrix} 1&0 \end{bmatrix} \varvec{x}_\mathrm {c}, \end{aligned}$$

where $\varvec{l}(\theta ^* - y)$ couples the observer to the t-pendulum through the observer gain vector $\varvec{l}=\left[ l_1,\; 0\right] ^\top$. The observer does not only filter out the undesired oscillation $\psi$, but also noise in the force measurement. An observer gain $l_1$ in the range of $\omega$ showed to yield a good compromise between fast transient behavior (large $l_1$) and noise filtering (small $l_1$). The smooth cartesian cart-pendulum states can then be transformed into polar states according to (10) and (15). The observer represents the abstract cart-pendulum dynamics (4) without inputs. Simulations and experiments showed that it suffices to use ${\hat{\omega }}$ as the estimate for the small angle approximation ${\hat{\omega }}_0$ needed in (31). We summarize these two steps as *projection onto the abstract cart-pendulum*.

#### Complete Control Law for the t-Pendulum

As suggested in [48], we do not directly command the acceleration in (12). Instead, we filter out remaining high frequency oscillations on the phase angle $\varphi$ through application of a second-order filter

32 $$\begin{aligned} G(s)=\frac{{\ddot{r}}_1}{r^\mathrm {d}_1}= & {} \frac{s^2\left( \frac{{\hat{\omega }}}{c_0}\right) ^2}{s^2+2\zeta \frac{{\hat{\omega }}}{c_0}s+\left( \frac{{\hat{\omega }}}{c_0}\right) ^2}, \end{aligned}$$

with design parameters $c_0$ and $\zeta$, to the reference trajectory

33 $$\begin{aligned} r^\mathrm {d}_1 = -\frac{a_1}{|G(j{\hat{\omega }})|}\sin (\varphi -\angle G(j{\hat{\omega }})). \end{aligned}$$

The acceleration results in

34 $$\begin{aligned} {\ddot{r}}_1&\simeq a_1 \;{\omega }^2 \frac{|G(j\omega )|}{|G(j {{\hat{\omega }}})|} \sin (\varphi -\angle G(j {{\hat{\omega }}})+ \angle G(j\omega )) \nonumber \\&\approx a_1 \;{{\omega }}^2 \sin (\varphi ). \end{aligned}$$

Hence, we make use of the sinusoidal shape of ${\ddot{r}}_1$ by including knowledge on the expected phase shift $\angle G(j{\hat{\omega }})$ and amplitude shift $|G(j{\hat{\omega }})|$ at ${\hat{\omega }}$. Use of position $r_1$ as a reference for the robot low-level controller circumvents drift. Furthermore, by imposing limits on $a_1$, the workspace of the robot can be limited [13, 48].

### Projection and Energy-Based Controller for the afa-System

#### Simple Pendulum-like Arm

Based on the results of [15], we model the robot end effector to behave as a cylindrical simple pendulum with human-like parameters of shoulder damping ${d_\rho }$, mass $m_\mathrm {a}$, length $l_\mathrm {a}$ and density $\varrho _\mathrm {a}$ for the experiments with a robotic manipulator in Sect. 8. The robot arm dynamics are

35 $$\begin{aligned} I_\rho {\ddot{\rho }} = - d_\rho {\dot{\rho }} + t_g - t_{f_{1}} + d_\psi {\dot{\psi }} + k_\psi \psi + t_{{\mathrm {s}},1}, \end{aligned}$$

where $I_\rho$ is the arm moment of inertia with respect to the shoulder and $t_g$ and $t_{f_{1}}$ are torques around the *z*-axis of coordinate system $\{ \mathrm {w}\}$ caused by gravity and the applied interaction forces at the wrist $\varvec{f}_{1}$, respectively. The wrist joint dynamics are

36 $$\begin{aligned} I_\psi (\ddot{\psi }+{\ddot{\rho }}) = -d_\psi {\dot{\psi }} - k_\psi \psi - t_{1z}, \end{aligned}$$

with moment of inertia $I_\psi$, damping $d_\psi$ and stiffness $k_\psi$. The *z*-component $t_{1z}$ of applied torque $\varvec{t}_1$ is measured at the interaction point with the flexible object.

#### Projection onto the Abstract Torque-Pendulum

We base the projection of the afa-system onto the abstract torque-pendulum on a simple summation $\theta ^*=\rho + \psi$ and the observer with simple pendulum dynamics in (31).

#### Complete Control Law for the afa-System

No additional filtering is applied for the computed shoulder torque. However, the wrist damping dissipates energy injected at the shoulder. The energy flow loss due to wrist damping is ${\dot{E}}_{d_\psi }=-d_\psi {\dot{\psi }}^2$. We approximate the injected energy flow at the shoulder as

37 $$\begin{aligned} {\dot{E}}_{t_{\mathrm {s},d\psi }}= t_{\mathrm {s},d_\psi } {\dot{\rho }} \approx a_{d_\psi } \vartheta _r {\hat{\omega }} \sin ^2\varphi \approx \frac{1}{2} a_{d_\psi } \vartheta _r {\hat{\omega }}, \end{aligned}$$

where we inserted $t_{\mathrm {s},d_\psi }=-a_{d_\psi }\sin \varphi$ according to (13), used ${\dot{\rho }} \mathop {=}\limits ^{{\dot{\rho }}\approx {\dot{\vartheta }}} -\vartheta _r {\hat{\omega }} \sin \varphi$ of (16) and approximated $\sin ^2\varphi$ by its mean. Setting ${\dot{E}}_{d_\psi } + {\dot{E}}_{t_{\mathrm {s},d\psi }}\mathop {=}\limits ^{!} 0$ yields amplitude factor $a_{d_\psi }{=\frac{2 d_\psi {\dot{\psi }}^2}{\vartheta _r {\hat{\omega }}} }$ for wrist damping compensation.

For the experiments, we add human-like shoulder damping $d_\rho$ to the passive arm behavior. During active follower or leader control the shoulder damping is compensated for by an additional shoulder torque of $t_{\mathrm {s},d_\rho }= d_\rho \; {\dot{\rho }}$. The complete control law results in

38 $$\begin{aligned} t_{\mathrm {s},1} = - a_1 \sin (\varphi ) + t_{\mathrm {s},d_\psi } + t_{\mathrm {s},d_\rho }. \end{aligned}$$

## Evaluation in Simulation

The linear fundamental dynamics (FD) derived in Sect. 4 enabled the design of adaptive leader and follower controllers in Sect. 5. However, the FD approximates the behavior of the abstract cart- and torque-pendulums, which represent the desired oscillations of the t-pendulum and the afa-system. In this section, we analyze the FD-based controllers in interaction with the abstract cart- and torque-pendulums with respect to stability of the $\omega$-estimation (Sect. 7.3), reference trajectory tracking (Sect. 7.4) and follower contribution (Sect. 7.5). For simplicity, we assume full state feedback $\varvec{x}_\mathrm {c}$ and use the variables $\theta _E$ and $\theta _E^\mathrm {d}$ also for the abstract cart- and torque-pendulums.

### Simulation Setup

The simulations were performed using *MATLAB/Simulink*. We modeled the cart-pendulum as a point mass $m_\mathrm {o}=10\,\hbox {kg}$ attached to a massless pole of length $l_\mathrm {o}=0.6\,\hbox {m}$. The torque-pendulum consisted of two rigidly attached cylinders with uniform mass distribution. The upper cylinder was of mass, density and length comparable to a human arm: $m_\mathrm {a}=3.35\,\hbox {kg}$ [7], $\varrho _\mathrm {a}=1100 \,\hbox {kg}/\hbox {m}^3$ [11], $l_\mathrm {a}=0.56\,\hbox {m}$ [15]. The lower cylinder had the same radius, but mass $m_\mathrm {o}=10\,\hbox {kg}$ and length $l_\mathrm {o}=0.4\,\hbox {m}$.

The following control gains stayed constant for all simulations $K_\mathrm {d}=0.4\,1/\hbox {s}$, $T_{\mathcal {F}}=1/\hbox {s}$, $c_0=0.9$, $\zeta =1.2$. We started all abstract cart- and torque-pendulum simulations with a small angle $\vartheta (t=0)=2^{\circ }$ and zero velocity ${\dot{\vartheta }}(t=0)=0\,\hbox {rad}/\hbox {s}$ in order to avoid initialization problems, e.g., of the phase angle $\varphi$.

### Measures

#### Analysis of Controller Performance

We analyzed the controller performance based on settling time $T_\mathrm {s}$, steady state error *e* and overshoot *o*. The settling time $T_\mathrm {s}$ was computed as the time after which the energy $\theta _E$ stayed within bounds $\pm \epsilon _\theta =\pm 8\,{\%}$ around the energetic steady state value ${\bar{\theta }}_E$. We defined the steady state error as $e={\theta }_E^\mathrm {d} - {\bar{\theta }}_E$ and the overshoot as $o=\mathrm {max}_t(\theta _E-\bar{\theta }_E)$.

#### Analysis of Effort Sharing

The energy flows to the abstract cart-pendulum were calculated based on velocities and applied force along the motion $\dot{E}_{1}=\frac{1}{2} {\dot{r}}_{1} f_{x}$, where $f_{x}=f_{1x}=f_{2x}$. The energy flows to the abstract torque-pendulum were calculated based on angular velocity and applied torque $\dot{E}_{1}=\frac{1}{2} {\dot{\vartheta }} \;t_{\mathrm {s,1}}$, where ${\dot{\vartheta }}={\dot{\vartheta }}_1={\dot{\vartheta }}_2$. The multiplication with $\frac{1}{2}$ reflects that the agents equally share the control over the abstract pendulums in (4) and (5).

We based the analysis of the effort sharing between the agents on the relative energy contribution of the follower $\varGamma _{\mathcal {F}}$. The definition in (2) is based on the time derivative of the oscillation amplitude ${\dot{\theta }}_{E,{\mathcal {F}}}$ and ${\dot{\theta }}_{E,{\mathcal {L}}}$, which requires use of the simple pendulum approximations. In order not to rely on approximations, we define the relative follower contribution

39 $$\begin{aligned} \varGamma _{\mathrm {in},{\mathcal {F}}}=\frac{\int _0^{T_\mathrm {s}}{{\dot{E}}_{\mathcal {F}} \mathrm {d}\tau }}{\int _0^{T_\mathrm {s}}{({\dot{E}}_{\mathcal {F}} + {\dot{E}}_{\mathcal {L}}) \mathrm {d}\tau }}. \end{aligned}$$

The above computation has the drawback that for mechanisms with high damping $\varGamma _{\mathrm {in},{\mathcal {F}}}<\varGamma _{{\mathcal {F}}}^\mathrm {d}$, because the follower reacts to changes in object energy and, thus, the leader accounts for damping compensation. Therefore, we define a second relative follower contribution based on the object energy *E* for comparison

40 $$\begin{aligned} \varGamma _{\mathrm {obj},{\mathcal {F}}}=\frac{\int _0^{T_\mathrm {s}}{{\dot{E}}_{\mathcal {F}} \mathrm {d}\tau }}{E(T_\mathrm {s}) }. \end{aligned}$$

For the abstract simple pendulums we use $E=E_\theta$. Note that $\varGamma _{\mathrm {obj},{\mathcal {F}}}+\varGamma _{\mathrm {obj},{\mathcal {L}}}\ne 1$ for a damped mechanism.

### Stability Limits of the $\omega$-Estimation

**Fig. 10:**
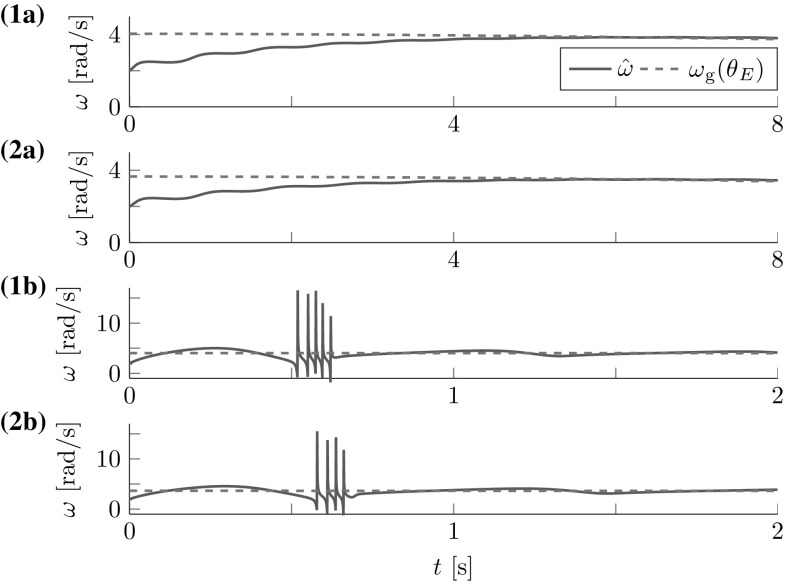
Natural frequency estimation for the (**1a–b**) cart-pendulum and (**2a-b**) torque-pendulum: (**a**) the estimate ${\hat{\omega }}$ smoothly approaches the geometric mean approximation of the natural frequency $\omega _g(\theta _E)$ for an estimation time constant $T_\omega =2\,\hbox {s}$, (**1b**) first signs of instability occur for $T_\omega =0.17\,\hbox {s}$ for the cart-pendulum and (**2b**) for $T_\omega =0.19\,\hbox {s}$ for the torque-pendulum. Note the different time and natural frequency scales. This result is in accordance with the theoretically found conservative stability bound $T_\omega > \max \left( \frac{1}{2 {\hat{\omega }}(t=0)},\frac{1}{2 \omega }\right)$ which evaluates to $T_\omega > 0.25\,\hbox {s}$ for ${\hat{\omega }}{(t=0)}=2\,\hbox {rad}/\hbox {s}$

The FD analysis in Sect. 5.1 revealed the theoretical stability bound (20). Here, we test its applicability to the cart- and torque-pendulums with energy dependent natural frequency $\omega$. Both lossless pendulums were controlled by one leader with constant amplitude factor $a{_{\mathcal {L}}}=0.04\,\hbox {m}$ for the cart-pendulum and $a{_{\mathcal {L}}}=5.5\,\hbox {Nm}$ for the torque-pendulum. The amplitude factors were chosen, such that for both pendulums approximately an energy level of $\theta _E\approx 60^{\circ }$ was reached after 8 s. Figure 10 shows the geometric mean approximation of the natural frequency $\omega _g(\theta _E)$ and the estimate ${\hat{\omega }}$ for two different time constants $T_\omega$ and ${{\hat{\omega }}(t=0)=2\,\hbox {rad}/\hbox {s}>0}$. The results support the conservative constraint found from the Lyapunov stability analysis in Sect. 5.1.

**Fig. 11:**
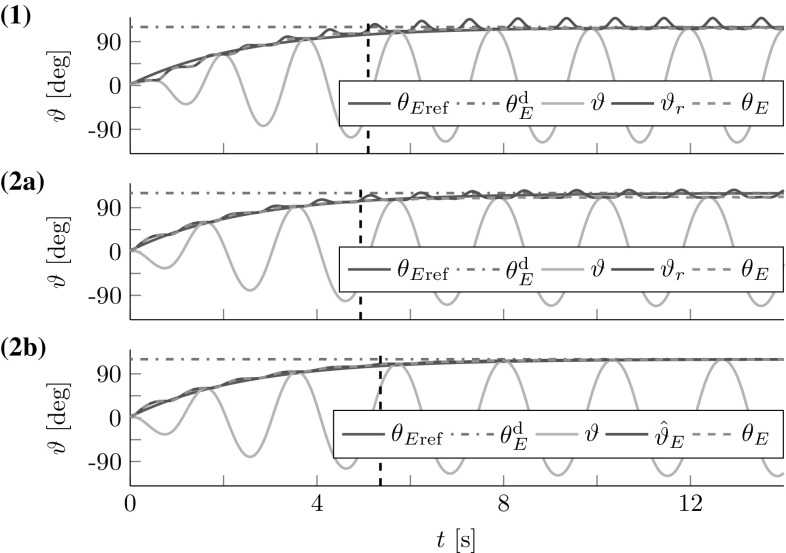
Reference dynamics tracking for (**1**) cart-pendulum and (**2a**) torque-pendulum based on energy equivalent $\vartheta _r$ with $e_{{\ddot{r}}}=2.7^{\circ }$ and $e_{t}=8.3^{\circ }$, respectively. Usage of an estimate ${\hat{\vartheta }}_E$ instead of $\vartheta _r$ reduces the steady state error for the torque-pendulum to $e_t=0.5^{\circ }$ (**2b**). *Vertical dashed lines* mark settling times $T_\mathrm {s}$

### Reference Dynamics Tracking

Here, we evaluate how well reference dynamics tracking is achieved for a single leader interacting with the cart- and torque-pendulums, thus $\varGamma _{\mathcal {L}}=1$. In order to focus on the reference dynamics tracking, we used the geometric mean $\omega _g(\theta _E)$ with exact $\omega _0$ in (9) as an accurate natural frequency estimate for the leader controller. We set $K_\mathrm {d}=0.4\,1/\hbox {s}$ and $\theta _E^\mathrm {d}=120^{\circ }$ 3. The results for the lossless pendulums are displayed in Fig. 11. The simulation results support the considerations made in Sect. 6.1.

**Fig. 12:**
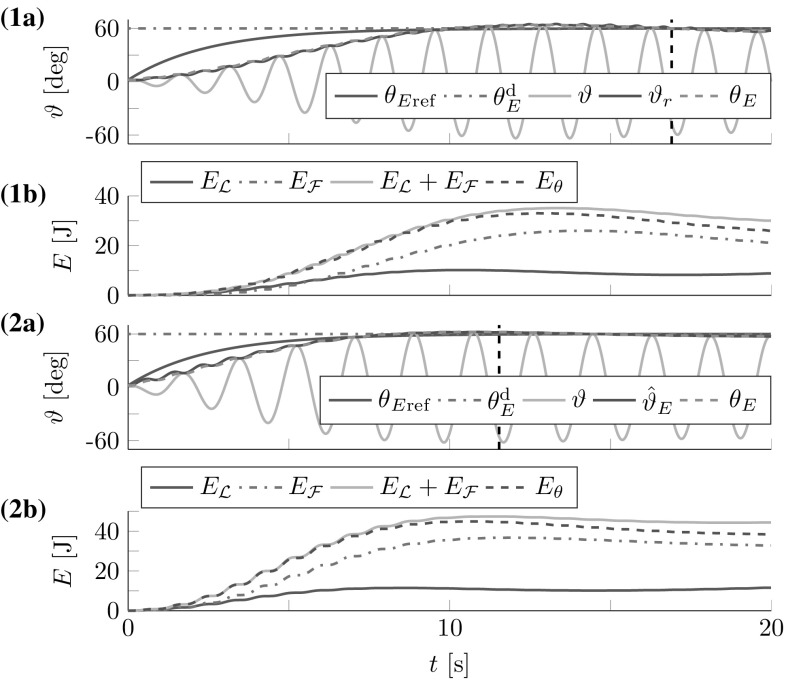
Simulated follower and leader interacting with the (1) abstract cart-pendulum and (2) abstract torque-pendulum for a desired relative follower contribution $\varGamma _{\mathcal {F}}^\mathrm {d}=0.7$: (**a**) angles and (**b**) energies. *Vertical dashed lines* mark settling times $T_\mathrm {s}$. The FD-based controllers allow for successful effort sharing

### Follower Contribution

For the follower contribution analysis, we ran simulations with a leader and a follower interacting with the abstract cart- and torque-pendulums for different desired relative follower contributions $\varGamma _{\mathcal {F}}^\mathrm {d}=0.3,0.5,0.7$. The pendulums were slightly damped with $t_{\mathrm {s},d_\rho }=-d_\mathrm {s}{\dot{\vartheta }}$ and $\frac{d_\mathrm {s}}{I_\vartheta }=0.01\,1/\hbox {s}$. The leader’s desired energy level was $\theta _E^\mathrm {d}=60^{\circ }$. In accordance with the stability analysis in Sect. 5.3, we initialized the $\omega$-estimation with ${\hat{\omega }}(t=0)=6\,\mathrm{rad}/\mathrm{s}>\omega$ for the abstract cart-pendulum and ${\hat{\omega }}(t=0)=2\,\hbox {rad}/\hbox {s}<\omega$ for the abstract torque-pendulum. The follower and leader controllers for the torque-pendulum made use of the approximation ${\hat{\vartheta }}_E$ in (29) instead of $\vartheta _r$ in (21) and (26).

The first three lines of Table 2 list the results for $\varGamma _{\mathcal {F}}^\mathrm {d} + \varGamma _{\mathcal {L}}^\mathrm {d} = 1$, including the relative follower contributions according to (39) and (40) and the overshoot *o*. Figure 12 shows angles and energies over time for the most challenging case of $\varGamma _{\mathcal {F}}^\mathrm {d}=0.7$. The damping resulted in increased steady state errors of $e_{{\ddot{r}}} =4.7^{\circ }$ for the abstract cart-pendulum and $e_{\tau } =2.5^{\circ }$ for the abstract torque-pendulum. The $\omega$-estimation and filtering for the energy flow estimate $\hat{{\dot{\vartheta }}}_r$ on the follower side caused a delay with respect to the reference dynamics $\theta _{E\mathrm {ref}}$. With respect to effort sharing, higher $\varGamma _{\mathcal {F}}^\mathrm {d}$ resulted in increased overshoot *o* (see Table 2). Successful effort sharing was achieved, with $\varGamma _{\mathcal {F}} \approx \varGamma _{\mathcal {F}}^\mathrm {d}$.

The last two lines of Table 2 list the results for $\varGamma _{\mathcal {F}}^\mathrm {d} + \varGamma _{\mathcal {L}}^\mathrm {d} \ne 1$. The results conform to the FD analysis in Sect. 5.3: $\varGamma _{\mathrm {in},{\mathcal {F}}} \approx \varGamma ^\mathrm {d}_{\mathcal {F}}\approx \varGamma _{\mathrm {obj},{\mathcal {F}}}$ with $\varGamma _{\mathrm {in},{\mathcal {L}}}=1-\varGamma _{\mathrm {in},{\mathcal {F}}}$. The transient behavior is predominantly influenced by $\varGamma _{{\mathcal {L}}}^\mathrm {d}$. Low (high) values $\varGamma _{\mathcal {F}}^\mathrm {d} + \varGamma _{\mathcal {L}}^\mathrm {d} <{(>)\;} 1$ yield slower (faster) convergence to the desired energy level with small (increased) overshoot *o*. An increased *o* comes along with increased transient behavior that settles only after $T_\mathrm {s}$. As a consequence, $\varGamma _{\mathrm {in},{\mathcal {F}}}$ and $\varGamma _{\mathrm {obj},{\mathcal {F}}}$ exceed $\varGamma _{\mathcal {F}}^\mathrm {d}$.

**Table 2:** Effort sharing results

$\varGamma _{\mathcal {F}}^\mathrm {d}/\varGamma _{\mathcal {L}}^\mathrm {d}$	Abstr. cart-pend.			Abstr. torque-pend.		
	$o[^{\circ }]$	$\varGamma _{\mathrm {in},{\mathcal {F}}}$	$\varGamma _{\mathrm {obj},{\mathcal {F}}}$	$o[^{\circ }]$	$\varGamma _{\mathrm {in},{\mathcal {F}}}$	$\varGamma _{\mathrm {obj},{\mathcal {F}}}$
0.3/0.7	0.9	0.27	0.27	0.1	0.33	0.33
0.5/0.5	3.2	0.45	0.47	1.1	0.52	0.54
0.7/0.3	8.7	0.75	0.84	4.9	0.78	0.82
0.3/0.3	0.1	0.30	0.32	0.1	0.31	0.33
0.7/0.7	9.6	0.81	0.87	6.5	0.86	0.90

## Experimental Evaluation

The simulations in Sect. 7 analyze the presented control approach for the abstract cart- and torque-pendu- lum. In this section, we report on the results of real world experiments with a t-pendulum and a flexible object which test the controllers in realistic conditions: noisy force measurements, non-ideal object and robot behavior and a human interaction partner. Online Resources 1 and 2 contain videos of the experiments.

**Fig. 13:**
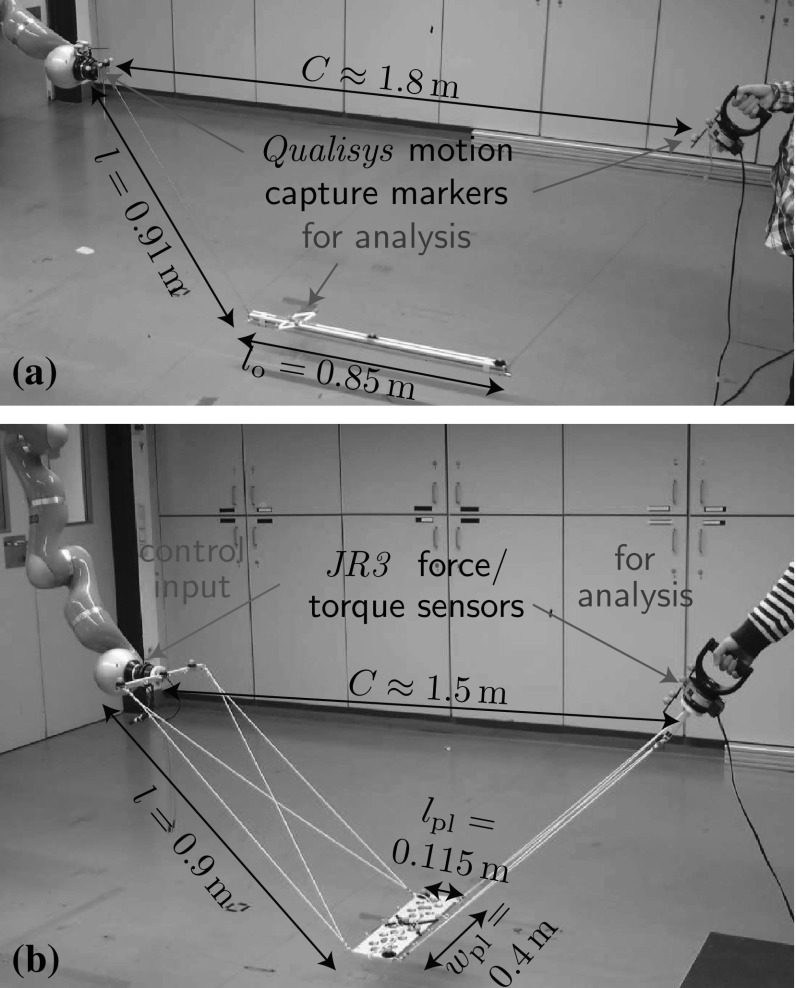
Experimental setups for (**a**) pendulum-like and (**b**) flexible object swinging: One side of the objects was attached to the end effector of a *KUKA LWR 4+* robotic manipulator under impedance control on joint level (joint stiffness 1500 Nm/rad and damping 0.7 Nm s/rad). The other side was attached to a handle that was either fixed to a table or held by the human interaction partner

### Experimental Setup

#### Hardware Setup

Figure 13 shows the experimental setups with pendulum-like and flexible objects. Due to the small load capacity of the robotic manipulator^4^, we used objects of relatively small mass $m_\mathrm {o}=1.25\,\hbox {kg}$ for the t-pendulum and $m_\mathrm {o}=1.61\,\hbox {kg}$ for the flexible object. The flexible object was composed of an aluminum plate connected to two aluminum bars through rubber bands. Such flexible object can be seen as an especially challenging object as it only loosely couples the agents and its high elasticity can cause unwanted oscillations.

#### Software Implementation

The motion capture data was recorded at 200 Hz and streamed to a *MATLAB/Simulink Real-Time Target* model. The Real-Time Target model was run at 1 kHz, received the force/torque data and contained the presented energy-based controller and the joint angle position controller of the robotic manipulator. For the analysis, we filtered the motion capture data and the force/torque data by a third-order butterworth low-pass filter with cutoff frequency 4 Hz.

The following control parameters were the same for all experiments $K_\mathrm {d}=0.4\,1/\hbox {s}$, $T_{\mathcal {F}}=1\,\hbox {s}$, $D_{\mathcal {F}}=1$, $c_0=0.9$, $\zeta =1.2$ and $l_1=3.6\,1/\hbox {s}$. The $\omega$-estimation used a time constant $T_\omega =2\,\hbox {s}$ and was initialized to ${\hat{\omega }}(t=0)=6\,\hbox {rad}/\hbox {s}$ for the t-pendulum. For the flexible object swinging, we controlled the robot to behave as a simple pendulum (see Sect. 6.3) with human arm parameters given in Sect. 7.1. The wrist parameters were $I_\psi =0.01\,\hbox {kg}\;\hbox {m}^2$, $d_\psi =4\,\hbox {Nm}\;\hbox {s}/\hbox {rad}$, $k_\psi =3\,\hbox {Nm}/\hbox {rad}$. The projected object length estimate needed for the approximation of the abstract torque-pendulum moment of inertia $\hat{I}_\vartheta$ was set to ${\hat{l}}^*_\mathrm {o}=0.64\,\hbox {m}$. The $\omega$-estimation used a time constant $T_\omega =4\,\hbox {s}$ and was initialized to ${\hat{\omega }}(t=0)=2\,\hbox {rad}/\hbox {s}$.

### Measures

We used the same measures to analyze the experiments as for the simulations in Sect. 7.2. Extensions and differences are highlighted in the following.

#### Analysis of the Projections onto the Abstract Cart- and Torque-Pendulums

Ideally, during steady state, the disturbance oscillations is close to zero $\psi \approx 0$, the abstract pendulum angle should be close to the actual object deflection $\vartheta \approx \theta$ and the energies should match $\vartheta _r \approx {\hat{\vartheta }}_E \approx \theta _E$. From motion capture data we obtained $\theta$ and for the t-pendulum $\psi$. The undesired oscillation of the afa-system is the known wrist angle $\psi$. From $\theta$, its numerical time derivative ${\dot{\theta }}$ and ${\hat{\omega }}_0$, the energy equivalent $\theta _E$ was computed.

#### Analysis of Effort Sharing

The energy flows of the agents were calculated based on ${\dot{E}}_{i} = \varvec{f}_{i}^\top \dot{\varvec{r}}_{i} + \varvec{t}_{i}^\top {\varvec{\varOmega }}_{i}$ with $i=1,2$, interaction point rotational velocities ${\varvec{\varOmega }}_{i}$ and $\varvec{t}_{i} \approx \varvec{0}$ for the t-pendulum. The energy contained in the object was calculated based on object height $y_\mathrm {o}$ and object twist $\dot{\varvec{\xi }}_\mathrm {o}=\left[ \dot{\varvec{r}}_\mathrm {o} ,\; {\varvec{\varOmega }}_\mathrm {o} \right] ^\top$

41 $$\begin{aligned} E=m_\mathrm {o} g y_\mathrm {o} + \frac{1}{2} \varvec{\dot{\xi }}_\mathrm {o}^\top \varvec{M}_\mathrm {o} \varvec{\dot{\xi }}_\mathrm {o}. \end{aligned}$$

The mass matrix $\varvec{M}_\mathrm {o} \in \mathbb {R}^{6\times 6}$ is composed of a $3\times 3$ diagonal matrix with the object mass $m_\mathrm {o}$ as diagonal entries and a $3\times 3$ moment of inertia tensor $\varvec{I}_\mathrm {o}$. The t-pendulum object moment of inertia $\varvec{I}_\mathrm {o}$ was approximated as a cylinder with uniform mass distribution of diameter $d_\mathrm {o}=0.05\,\hbox {m}$. For the afa-system, we neglected energy contained in the rubber bands and the aluminum bars attached to the force/torque sensors and computed the energy contained in the aluminum plate of mass $m_\mathrm {pl}=1.15\,\hbox {kg}$ and thickness $h_\mathrm {pl}=0.012\,\hbox {m}$ under the simplifying assumption of uniform mass distribution (see Fig. 13 for further dimensions). Above variables are expressed in a fixed world coordinate system translated such that $y_\mathrm {o}=0\,\hbox {m}$ for $\theta =\psi =0^{\circ }$. The energy contained in undesired system oscillations $\psi$ can be approximated as $E_\psi \approx E-E_\theta$.

### Experimental Controller Evaluation for the t-Pendulum

We present results for three t-pendulum experiments: maximum achievable energy (Sect. 8.3.1), active follower contribution (Sect. 8.3.2) and excitation of undesired $\psi$-oscillation (Sect. 8.3.3).

#### Maximum Achievable Energy (Robot Leader and Passive Human)

The limitations of the controller with respect to the achievable energy levels were tested with a robot leader $\mathrm {A1=R}={\mathcal {L}}$. A human passively held the handle of agent $\mathrm {A2=H=P}$ in order to avoid extreme $\psi$-oscilla- tion excitation at high energy levels due to a rigid fixed end. The t-pendulum started from rest ($\theta _E(t=0)\approx \psi _E(t=0)\approx 0$). The desired energy level $\theta _E^\mathrm {d}$ was incrementally increased from 15 deg to 90 deg. The desired relative energy contribution of the robot was $\varGamma ^\mathrm {d}_\mathrm {R}=1$.

**Fig. 14:**
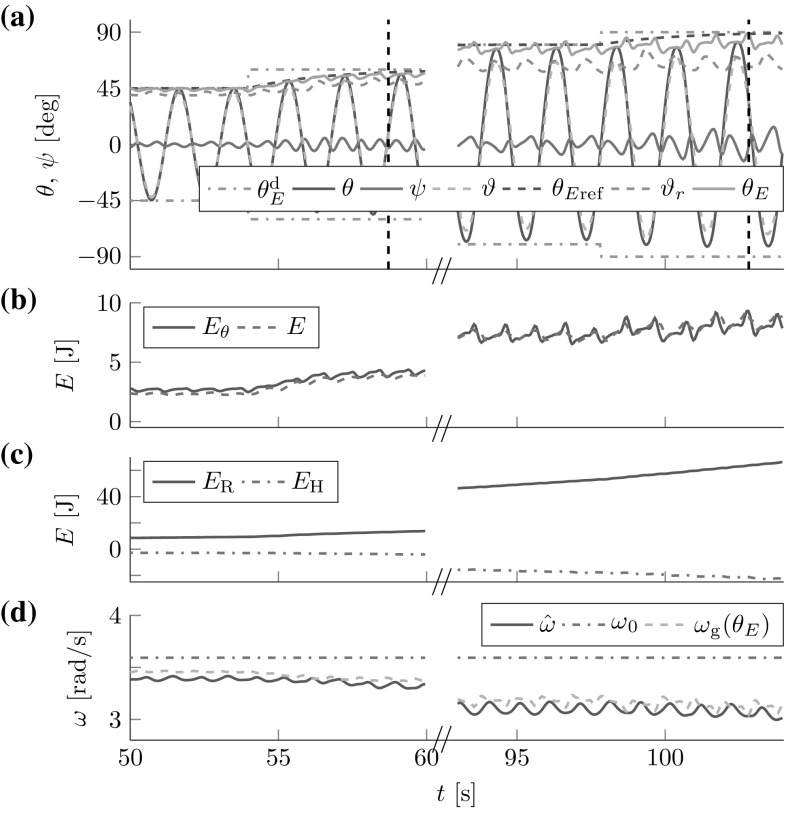
Maximum achievable energies $\theta _E^\mathrm {d}$ for the t-pendulum: (**a**) deflection angles and energy equivalents, (**b**) energies contained in the t-pendulum and (**c**) contributed by the human and the robot (**d**) natural frequency estimates. *Vertical dashed lines* mark settling times $T_\mathrm {s}$. A robot leader can reach deflection angles $\theta > 80^{\circ }$ in interaction with a passive human

The robot successfully controlled the t-pendulum energy to closely follow the desired reference dynamics (see Fig. 14).

The steady state error increased with higher desired energy due to increased damping, e.g., $e=0.4^{\circ }$ at $\theta _E^\mathrm {d}=15^{\circ }$ and $e=8.2^{\circ }$ at $\theta ^\mathrm {d}_E=90^{\circ }$. The energy contained in the undesired oscillation increased from $\psi _E=1.4^{\circ }$ at $\theta _E^\mathrm {d}=15^{\circ }$ to $\psi _E=15.6^{\circ }$ at $\theta _E^\mathrm {d}=90^{\circ }$ and was, thus, kept in comparably small ranges. With increased $\psi$-oscillation, the t-pendulum behaves less simple pendulum-like, which also becomes apparent in an increased difference between $\vartheta _r$ and $\theta _E$. The successful reference dynamics tracking and close estimate $\vartheta _r\approx \theta _E$ for smaller and intermediate energy levels and the close $\omega$-estimation support the applicability of the fundamental dynamics (FD)-based leader controller.

#### Active Follower Contribution (Robot Follower and Human Leader)

A robot follower $\mathrm {A1=R}={\mathcal {F}}$ with $\varGamma ^\mathrm {d}_\mathrm {R}=0.5$ interacted with a human leader $\mathrm {A2=H}={\mathcal {L}}$. The t-pendulum started from rest ($\theta _E(t=0)\approx \psi _E(t=0)\approx 0$). The human leader was asked to first inject energy to reach $\theta ^\mathrm {d}_E=60^{\circ }$, to hold the energy constant and finally to release the energy from the pendulum again. The desired energy limit was displayed to the human via stripes of tape on the floor to which the pendulum mass had to be aligned to at maximum deflection angles.

**Fig. 15:**
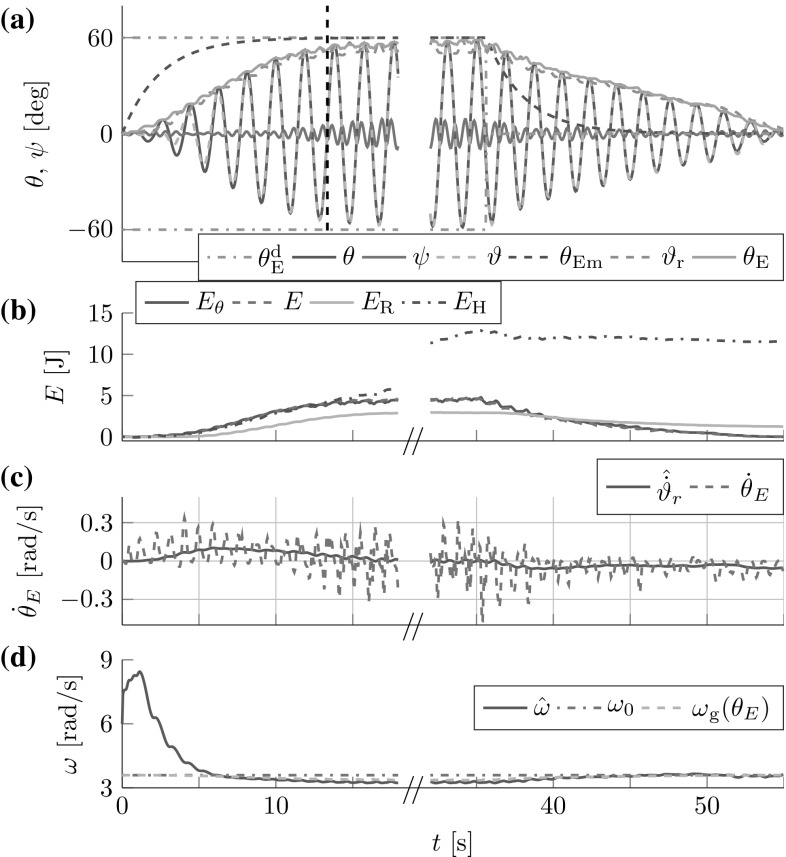
Robot follower cooperatively injecting energy into the t-pendulum with a human leader: (**a**) deflection angles and energy equivalents, (**b**) energies contained in the t-pendulum and contributed by the human and the robot, (**c**) actual and estimated energy flows, (**d**) natural frequency estimates. The *vertical dashed line* marks settling time $T_\mathrm {s}$

The human–robot team successfully injected energy until $\theta _E^\mathrm {d}=60^{\circ }$ was reached with $e = 3^{\circ }$ (see Fig. 15). Similar to the simulations, the reference dynamics were tracked with a delay. The undesired oscillation increased, but did not exceed $\psi _{E}=10.4^{\circ }$. The object energy flow ${\dot{\theta }}_E$ highly oscillated, which is in accordance with the results from human–human rigid object swinging [15]. The robot successfully detected and imitated the object energy flow. During the 20 s constant energy phase, the human compensated for energy loss due to damping. The relative energy contributions $\varGamma _{\mathrm {Rin}}=0.35$ and $\varGamma _{\mathrm {Robj}}=0.57$ were close to the desired $\varGamma _\mathrm {R}^\mathrm {d}=0.5$. The follower controller highly depends on the FD approximation. Thus, the successful energy sharing between a human leader and a robot follower further supports the efficacy of the FD-based controllers to human–robot dynamic object manipulation.

#### Excitation of Undesired $\psi$-Oscillation (Robot Leader and Fixed End)

The pendulum mass was manually released in a pose with high initial $\psi$-oscillation $\psi _E(t=0)=29^{\circ }$, but $\theta _E(t=0)\approx 0$. A goal energy of $\theta ^\mathrm {d}_E=40^{\circ }$ was given to the robot leader $\mathrm {A1=R}={\mathcal {L}}$ with $\varGamma ^\mathrm {d}_\mathrm {R}=1$, while the handle of agent $\mathrm {A}2=0$ was fixed.

**Fig. 16:**
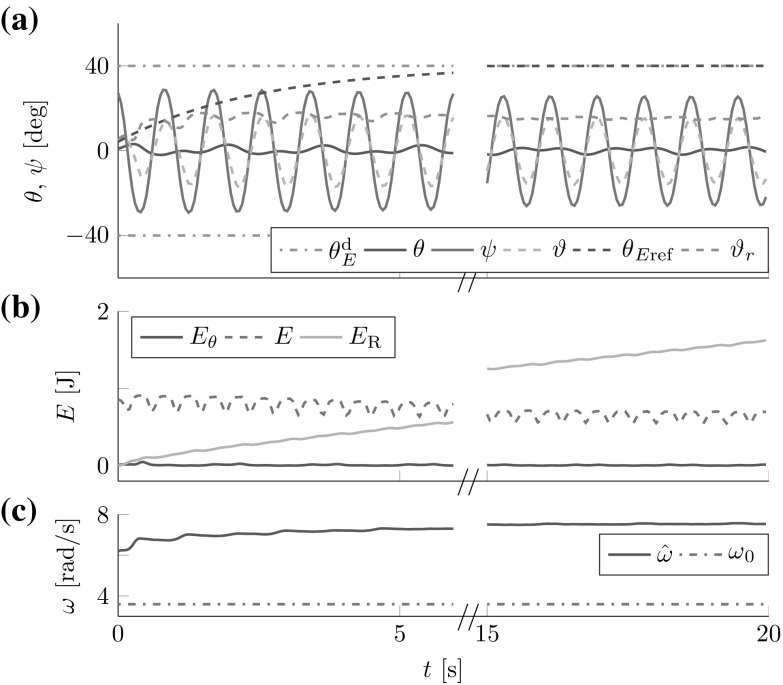
Strong initial $\psi _E$ for robot leader and fixed end: (**a**) deflection angles and energy equivalents, (**b**) energies contained in the t-pendulum and contributed by the robot, (**c**) natural frequency estimates. The robot detected the natural frequency of the less simple pendulum-like $\psi$-oscillation and sustained it

The robot identified the natural frequency of the $\psi$-oscillation and tried to inject energy to reach the desired amplitude of $\theta _E^\mathrm {d}=40^{\circ }$ (see Fig. 16). Thus, the robot failed to excite the desired $\theta$-oscillation and keep unwanted oscillations in small bounds as defined in Sect. 3. However, considering the controller implementation given in Fig. 8, this experimental result supports the correct controller operation: the $\omega$-estimation identified the frequency of the current oscillation, here the undesired $\psi$-oscillation. Based on ${\hat{\omega }}$, the leader controller was able to inject energy into the $\psi$-oscillation; not enough to reach the desired amplitude of $\theta _E^\mathrm {d}=40^{\circ }$, but enough to sustain the oscillation. Note that the $\psi$-oscillation is highly damped, less simple pendulum-like and in general more difficult to excite than the $\theta$-oscillation. Experiments with a controller that numerically differentiates the projected deflection angle $\theta ^*$, instead of using the observer, less accurately timed the energy injection. The result was a suppression of the $\psi$-oscillation through natural damping until the $\theta$-oscillation dominated ${\hat{\omega }}$ and $\theta _E^\mathrm {d}$ was reached.

On the one hand side, this experiment supports the control approach by showing that the controller is able to excite also less simple pendulum-like oscillations. On the other hand side, this experiment reveals the need for a higher level entity to detect failures as when the wrong oscillation is excited (see the discussion in Sect. 9.1).

### Experimental Controller Evaluation for the afa-System

Joint velocity limitations of the KUKA LWR restricted us to energies $\theta _E^\mathrm {d} \le 30^{\circ }$ for the afa-system experiments. We present experiments that investigate the maximum achievable energy (Sect. 8.4.1) and active follower contribution (Sect. 8.4.2).

**Fig. 17:**
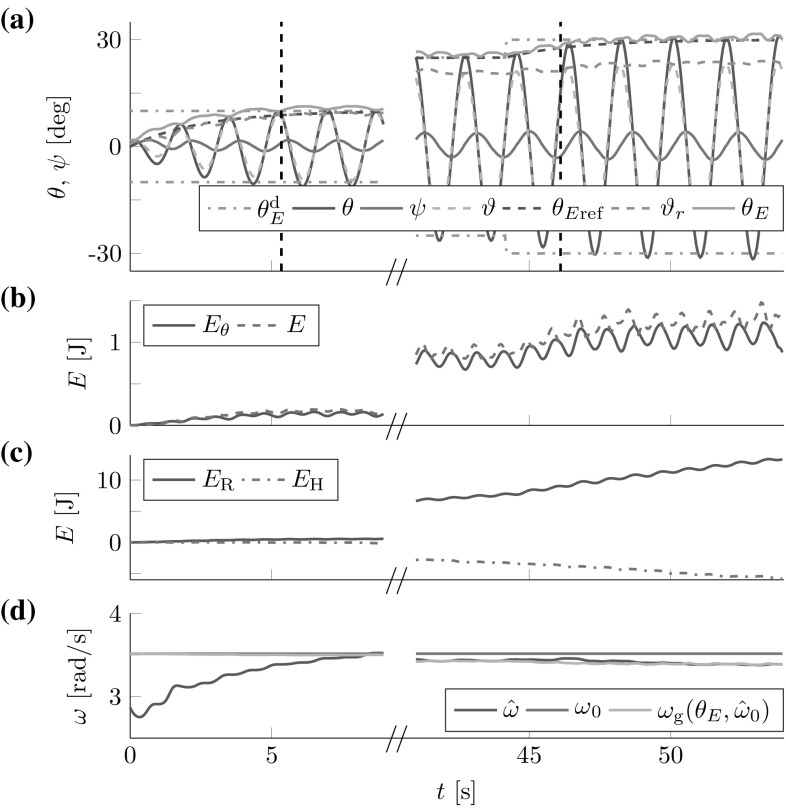
Maximum achievable energies are limited to $\theta _E = 30^{\circ }$ for the afa-system, due to joint velocity limits: (**a**) deflection angles and energy equivalents, (**b**) energies contained in the flexible object and (**c**) contributed by the human and the robot, (**d**) natural frequency estimates. *Vertical dashed lines* mark settling times $T_\mathrm {s}$

#### Maximum Achievable Energy (Robot Leader and Passive Human)

A robot leader $\mathrm {A1=R}={\mathcal {L}}$ interacted with a passive human leader $\mathrm {A2=H=P}$ under the same conditions as for the t-pendulum in Sect. 8.3.1. We incrementally increased $\theta _E^\mathrm {d}$ from 10 deg to 30 deg.

The robot leader closely followed the desired reference dynamics and achieved small steady state errors, e.g., $e=-0.9^{\circ }$ at $\theta _E^\mathrm {d}=10^{\circ }$ and $e=-0.6^{\circ }$ at $\theta _E^\mathrm {d}=30^{\circ }$ (see Fig. 17). Undesired oscillations at the wrist stayed below $\psi _E<4.3^{\circ }$. The projection of the flexible object onto the abstract torque-pendulum was performed based on the sum $\theta ^*=\psi + \rho$ and the simple pendulum observer. From Fig. 4 it seems like the sum $\psi + \rho$ overestimates the deflection angle at the shoulder. However, the known wrist angle $\psi$ only reflects the orientation of the flexible object at the robot interaction point. The flexibility of the object caused greater deflection angles $\theta$. Consequently, the abstract torque-pendulum energy equivalent $\vartheta _r$ closely followed the energy equivalent $\theta _E$ at small energies, but underestimated $\theta$ for increased energies. Nevertheless, the results are promising as they show that a controlled swing-up was achieved based on the virtual energy $\vartheta _r$ of the abstract torque-pendulum.

**Fig. 18:**
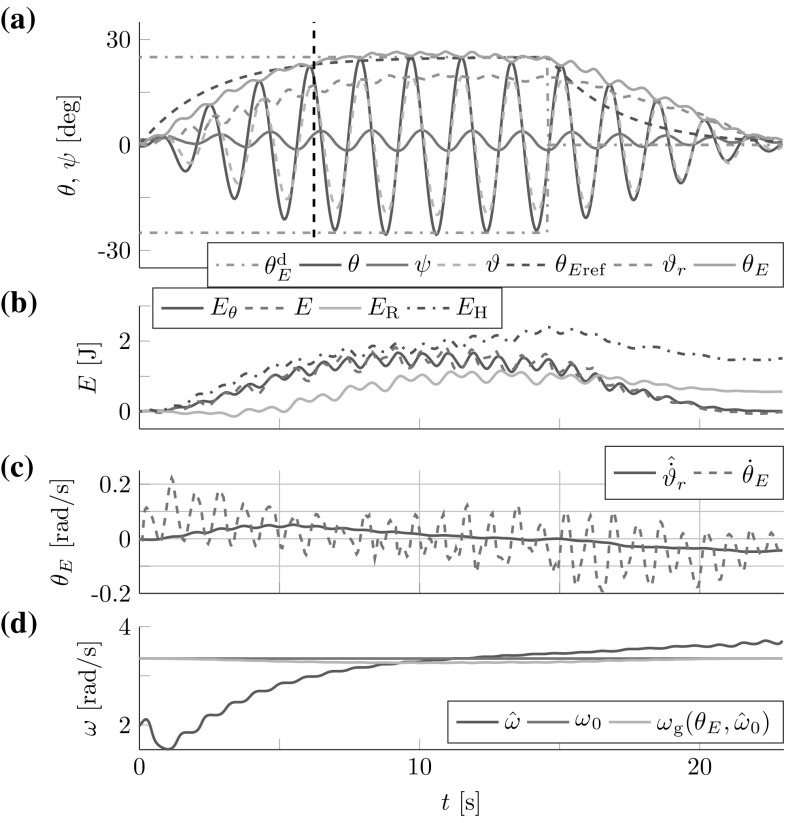
Robot follower cooperatively injecting energy into the flexible object with a human leader: (**a**) deflection angles and energy equivalents, (**b**) energies contained in the flexible object and contributed by the human and the robot, (**c**) actual and estimated energy flows, (**d**) natural frequency estimates. The energy contributions of the robot and the human show similar characteristics. The *vertical dashed line* marks settling time $T_\mathrm {s}$

#### Active Follower Contribution (Robot Follower and Human Leader)

A robot follower $\mathrm {A1=R}={\mathcal {F}}$ interacted with a human leader $\mathrm {A2=H}={\mathcal {L}}$ under the same conditions as for the t-pendulum in Sect. 8.3.2. Due to the hardware limitations we used $\theta ^\mathrm {d}_E=25^{\circ }$, but chose a higher and thus more challenging desired relative energy contribution of the robot follower of $\varGamma ^\mathrm {d}_\mathrm {R}=0.65$.

The robot successfully imitated the object energy flow, which led to human–robot cooperative energy injection to $\theta _E^\mathrm {d} = 25^{\circ }$ with small $e = -0.9^{\circ }$ (see Fig. 18). The human first injected energy into the passive robot arm which is equivalent to the robot initially withdrawing some energy from the object, before the robot can detect the object energy increase. Therefore and due to the filtering for $\hat{\dot{\vartheta }}_r$, the follower achieved only $\varGamma _{\mathrm {Rin}}=0.22$ and $\varGamma _{\mathrm {Robj}}=0.34$, when evaluated at $T_\mathrm {s}$. However, the relative follower contribution increased and reached, e.g., $\varGamma _{\mathrm {Rin}}=0.35$ and $\varGamma _{\mathrm {Robj}}=0.62$ at $t=11 \,\hbox {s}$. Interestingly, the energy contribution of the human and the robot were of similar shape, both for a robot follower and a robot leader. Thus, the simple pendulum-like behavior of the robot end effector allows to replicate human whole-arm swinging characteristics.

## Discussion

### Embedding of Proposed Controllers in a Robotic Architecture

One of the major goals of robotics research is to design robots that are able to manipulate unknown objects in a goal-directed manner without prior model knowledge or tuning. Robot architectures are employed to manage such complex robot functionality [42]. These architectures are often organized in three layers: the lowest layer realizes behaviors which are coordinated by an intermediate executive layer based on a plan provided by the highest layer. In this work, our focus is on the lowest layer: the behavior of cooperative energy injection into swinging motion, which is challenging in itself due to the underactuation caused by the multitude of DoFs of the pendulum-like and flexible objects. On the behavioral layer, we use high-frequency force and torque measurements to achieve continuous energy injection and robustness with respect to disturbances. The controllers presented implement the distinct roles of a leader and a follower. As known from human studies, humans tend to specialize, but do not rigidly stick to one role and continuously blend between leader and follower behaviors [40]. Role mixing or blending would be triggered by the executive layer. The executive layer would operate at a lower frequency and would have access to additional sensors as, e.g., a camera that allows to monitor task execution. Based on the additional sensor measurements, exceptions could be handled (e.g., when a wrong oscillation degree of freedom is excited as in Sect. 8.3.3), the required swinging amplitude $\theta _E^\mathrm {d}$ could be set and behavior switching could be triggered (e.g., from the object swing-up behavior to an object placement behavior).

Furthermore, additional object specific parameters could be estimated on the executive layer, as, e.g., damping or elastic object deformation. The fundamental dynamics (FD) approach does not model damping, and consequently $\varGamma _{{\mathrm {Robj}}}\approx \varGamma ^\mathrm {d}_\mathrm {R}$ indicates that the controller exhibits the desired behavior. However, that also means that $\varGamma _{{\mathrm {Rin}}} < \varGamma ^\mathrm {d}_\mathrm {R}$, because the leader compensates for damping. As all realistic objects exhibit non negligible damping, an increased robot contribution during swing-up can be achieved by increasing $\varGamma ^\mathrm {d}_\mathrm {R}$. The desired relative energy contribution $\varGamma ^\mathrm {d}_\mathrm {R}$ could thus serve as a single parameter that could, for instance, be adjusted online by the executive layer to achieve a desired robot contribution to the swing-up. Alternatively to an executive layer, a human partner could adjust a parameter as $\varGamma ^\mathrm {d}_\mathrm {R}$ online to achieve desired robot follower behavior and could also assure excitation of the desired oscillation.

### Generalizeability

The main assumption made in this work is that the desired oscillation is simple pendulum-like. Based on this assumption, the proposed approach is generalizable in the sense that it can be directly applied to the joint swing-up of unknown objects without parameter tuning^5^ (see video with online changing flexible object parameters in Online Resource 2). We regard the case of a robotic follower interacting with a human leader as an interesting and challenging scenario and therefore presented our method from the human–robot cooperation perspective. Nevertheless, the proposed method can also directly be employed for robot-robot teams or single robot systems as, e.g., quadrotors and can also be used to damp oscillations instead of exciting them. The task of joint energy injection into a flexible bulky object might appear to be a rare special case. However, it is a basic dynamic manipulation skill that humans possess and should be investigated in order to equip robots with universal manipulation skills.

We see the main take away message for future research from this work in the advantage of an understan- ding of the underlying FD. Based on the FD that encodes desired behavior, simple adaptive controllers can be designed and readily applied to complex tasks even when task parameters change drastically, as, e.g., when objects of different dimensions have to be manipulated.

### Dependence of Robot Follower Performance on the Human Interaction Partner

Performance measures as settling time $T_\mathrm {s}$ and steady state error *e* strongly depend on the behavior of the human partner. The robot follower is responsible for the resultant effort sharing. Ideally, the robot follower contributes with the desired fraction to the current change in object energy at all times $\dot{\vartheta }_{r,\mathrm {R}} = \varGamma ^\mathrm {d}_\mathrm {R} \dot{\vartheta }_r$. Necessary filtering and the approximations made by the FD do result in a delayed follower response and deviation from $\varGamma ^\mathrm {d}_\mathrm {R}$. However, for the follower, we do not make any assumptions on the way how humans inject energy into the system, e.g., we do not assume that human leaders follow the desired reference dynamics that we defined for robot leaders. This is in contrast to our previous work [13], where thresholds were tuned with respect to human swing-up behavior and the follower required extensive model knowledge to compute the energy contained in the oscillation. For demonstration purposes, we aimed for a smooth energy injection of the human leader for the experiments presented in the previous section. Energy was not injected smoothly to match modeled behavior, but only to enable the use of measures as the relative energy contribution at the settling time for effort sharing analysis.

### Alternatives to Energy-Based Swing-Up Controllers

Energy-based controllers as [48] are known to be less efficient than, e.g., model predictive control (MPC)-based controllers [31]. MPC can improve performance with respect to energy and time needed to reach a desired energy content. However, in this work, we do not aim for an especially efficient robot controller, but for cooperative energy injection into unknown objects. Use of MPC requires a model, including accurate mass and moment of inertia properties. Use of the energy-based controller of [48] allows to derive the FD as an approximate model. The FD reduces the unknowns to the natural frequency $\omega$ and moment of inertia estimate $I_\vartheta$ for the afa-system, which can be estimated online. Design of a follower controller is only possible, because the FD allows for a comparison of expectation to observation. How to formulate the expectation for an MPC-based approach is unclear and would certainly be more involved. The great advantage of the FD -based approach lies in its simplicity.

### Alternative Parameter Estimation Approaches

In this work, the goal of a leader controller is to track desired reference dynamics. Such behavior could also be achieved by employing model reference adaptive control (MRAC) [2] or by employing filters to compare applied amplitude factors *a* to the achieved energy increase to estimate the unknown FD parameter *B*. The disadvantage of MRAC and other approaches is that they need to observe the system energy $\vartheta _r$ online to estimate the system constant *B*. Having more than one agent interacting with the system does not only challenge the stability properties of MRAC, but also makes it impossible to design a follower that requires ${\hat{B}}$ to differentiate between its own and external influence on $\vartheta _r$.

The FD approximates the system parameter *B* by its mean, while the true value oscillates. The mean parameter *B* depends on the natural frequency $\omega$, which can be approximated by observing the phase angle $\varphi$. Because the FD states $\vartheta _r$ and $\varphi$ are approximately decoupled, reference dynamics tracking and energy flow imitation can be achieved for unknown objects.

The natural frequency $\omega$ could also be estimated by observing the time required by a full swing. Decrease of the observation period yields the continuous simple low-pass filter used in this article. Alternatively, the desired circularity of the phase space could be used to employ methods such as gradient descent [37] or Newton Raphson to estimate $\omega$. We chose the presented approach for its continuity and simplicity, as well as its stability properties with respect to the FD assumption.

### Stability of Human–Robot Object Manipulation

We proved global stability of the presented control approach for the linear FD. Stability investigations of the human–robot flexible object manipulation face several challenges. Firstly, dynamic models of the complex t-pendulum and afa-system would be required. Furthermore, the human interaction partner acts as a non-autonomous and non-reproducible system that is difficult to model and whose stability cannot be analyzed based on common methods [5]. In [23], Hogan presents results that indicate that the human arm exhibits the impedance of a passive object; however, this result cannot be directly applied to show stabilization of limit cycles, as the simple pendulum oscillation in this work, for a passivity-based stability analysis [24]. A stability analysis of the simpler, but nonlinear abstract simple pendulums requires a reformulation of the system dynamics in terms of the errors $\varDelta {\hat{\omega }}=\omega -{\hat{\omega }}$ and $\varDelta \vartheta _E=\vartheta _E^\mathrm {d}- \vartheta _E$. The lack of analytic solutions for $\omega (\vartheta _E)$ [6] and $\vartheta (\vartheta _E,\varphi )$ (see Sect. 4.4) impede the derivation of above error dynamics.

As our final goal is cooperative dynamic human–robot interaction, we refrained from further stability investigations in this paper and focused on simulation- and experiment-based analyses. The simulations and human–robot experiments suggest that the domain of attraction of the presented FD-based controllers is sufficiently large to allow for cooperative energy injection into nonlinear high energy regimes.

## Conclusions

This article presents a control approach for cooperative energy injection into unknown flexible objects as a first step towards human–robot cooperative dynamic object manipulation. The simple pendulum-like nature of the desired swinging motion allows to design adaptive follower and leader controllers based on simple pendulum closed-loop fundamental dynamics (FD). We consider two different systems and show that their desired oscillations can be approximated by similar FD. Firstly, a pendulum-like object that is controlled via acceleration by the human and the robot. Secondly, an oscillating entity composed of the agents’ arms and a flexible object that is controlled via torque at the agents’ shoulders. The robot estimates the natural frequency of the system and controls the swing energy as a leader or follower from haptic information only. In contrast to a leader, a follower does not know the desired energy level, but actively contributes to the swing-up through imitation of the system energy flow. Experimental results showed that a robotic leader can track desired reference dynamics. Furthermore, a robot follower actively contributed to the swing-up effort in interaction with a human leader. High energy levels of swinging amplitudes greater than $80^{\circ }$ were achieved for the pendulum-like object. Although joint velocity limits of the robotic manipulator restricted swinging amplitudes to $30^{\circ }$ for the “arm—flexible object—arm” system, the experimental results support the efficacy of our approach to human–robot cooperative swinging of unknown flexible objects.

In future work, we want to take a second step towards human–robot cooperative dynamic object manipulation by investigating controlled object placement as the phase following the joint energy injection. Furthermore, we are interested in applying the presented technique of approximating the desired behavior by its FD to different manipulation tasks.

### Electronic supplementary material

Below is the link to the electronic supplementary material.

Supplementary material 1 (mp4 37760 KB)

Supplementary material 2 (mp4 36382 KB)

## Figures and Tables

**Fig. 19:**
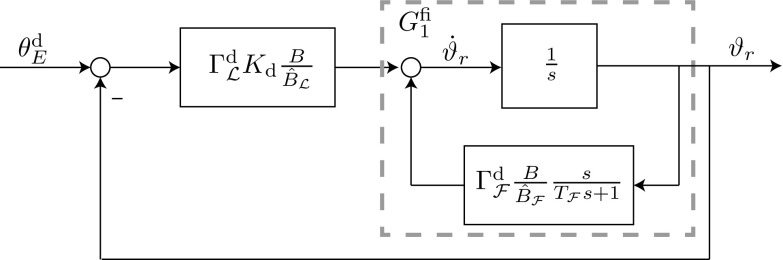
Rearranged block diagram for the computation of the transfer function $G^\mathrm {fi}(s)$: $\vartheta _r(s)=G^\mathrm {fi}(s) \theta _E^\mathrm {d}(s)$

**Fig. 20:**
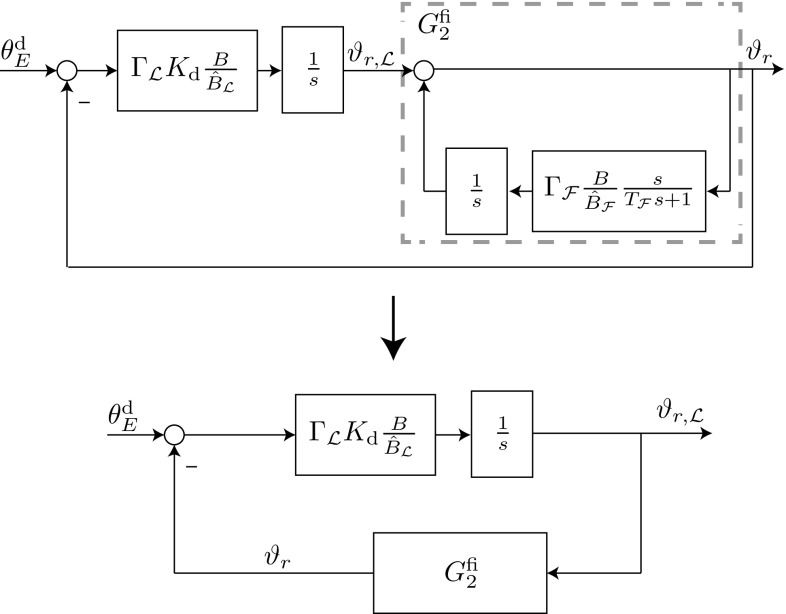
Rearranged block diagram for the computation of the energy contributed by the leader $\vartheta _{r,{\mathcal {L}}}=G_{{\mathcal {L}}}^\mathrm {fi}(s) \theta _E^\mathrm {d}(s)$

## Derivation of the Fundamental Dynamics

Application of the following three steps yields the dynamics of the abstract cart- and torque-pendulums (4), (5) in terms of the polar states $\varvec{x}_\mathrm {p}$:

1. Differentiation of (10) and (15) with respect to time
2. Insertion of the cartesian state dynamics (4) and (5)
3. Substitution of remaining cartesian states through polar states (16)

Step S1 applied to the phase angle $\varphi$ requires the time derivative of the ${{\mathrm{atan2}}}$-function, which is

42 $$\begin{aligned} {\frac{\mathrm {d}}{\mathrm {d}t} {{\mathrm{atan2}}}(y,x)= \frac{-y \frac{\mathrm {d}x}{\mathrm {d}t} + x \frac{\mathrm {d}y}{\mathrm {d}t}}{x^2 + y^2}.} \end{aligned}$$

We get

43 $$\begin{aligned} {\dot{\varphi }}\mathop {=}\limits ^{\mathrm {S1}}&\frac{\varOmega {\dot{\vartheta }}^2 -\varOmega \vartheta {\ddot{\vartheta }}}{\varOmega ^2\vartheta ^2+{\dot{\vartheta }}^2}\nonumber \\ \mathop {=}\limits ^{\mathrm {S2}}&\frac{\varOmega {\dot{\vartheta }}^2+\varOmega \omega _0^2 \vartheta \sin \vartheta -\varOmega \vartheta A}{\varOmega ^2\vartheta ^2+{\dot{\vartheta }}^2}\nonumber \\ \mathop {=}\limits ^{\mathrm {S3}}&\varOmega \sin ^2\varphi +\frac{\omega _0^2}{\varOmega \vartheta _{r}}\cos \varphi \sin (\vartheta _{r}\cos \varphi )\nonumber \\&-\frac{1}{\varOmega \vartheta _r} \cos \varphi A, \end{aligned}$$

44 $$\begin{aligned} {\dot{\vartheta }}_{r}\mathop {=}\limits ^{\mathrm {S1}}&\frac{\varOmega ^2\vartheta {\dot{\vartheta }} +{\dot{\vartheta }}{\ddot{\vartheta }}}{\varOmega \sqrt{\varOmega ^2\vartheta ^2 +{\dot{\vartheta }}^2}}\nonumber \\ \mathop {=}\limits ^{\mathrm {S2}}&\frac{\varOmega ^2\vartheta {\dot{\vartheta }} -\omega _0^2{\dot{\vartheta }}\sin \vartheta +{\dot{\vartheta }} A}{\varOmega \sqrt{\varOmega ^2\vartheta ^2+{\dot{\vartheta }}^2}}\nonumber \\ \mathop {=}\limits ^{\mathrm {S3}}&-\varOmega \vartheta _{r} \sin \varphi \cos \varphi +\frac{\omega _0^2}{\varOmega }\sin \varphi \sin (\vartheta _{r}\cos \varphi ) \nonumber \\&-\frac{1}{\varOmega }\sin \varphi A, \end{aligned}$$

with actuation terms $A=A_{{\ddot{r}}}$ for the abstract cart-pendulum

45 $$\begin{aligned} A_{{\ddot{r}}} \mathop {=}\limits ^{\mathrm {S3}}-\frac{\omega _{0}^2}{g} \cos (\vartheta _r \cos \varphi ) \frac{{\ddot{r}}_{{1}}+{\ddot{r}}_{{2}}}{2} \end{aligned}$$

and $A=A_{t}$ for the abstract torque-pendulum

46 $$\begin{aligned} A_{t}= & {} \frac{1}{I_\vartheta } \frac{t_{\mathrm {s},1}+t_{\mathrm {s},2}}{2}. \end{aligned}$$

The resultant state space representations are control affine and coupled

47 $$\begin{aligned} {\dot{\varvec{x}}}_\mathrm {p} = \varvec{f}_\mathrm {p}(\varvec{x}_\mathrm {p})+\varvec{g}_\mathrm {p} (\varvec{x}_\mathrm {p})u, \end{aligned}$$

with control input $u:=A$.

Insertion of the control laws (12) and (13) into $A=A_{{\ddot{r}}}$ and $A=A_{t}$ in (47) yield the state space representations with new inputs $a_{1}$ and $a_{2}$ of the form

48 $$\begin{aligned} {\dot{\varvec{x}}}_\mathrm {p} = \varvec{f}_\mathrm {p}(\varvec{x}_\mathrm {p})+{^a{\varvec{g}}}_\mathrm {p} (\varvec{x}_\mathrm {p}) \frac{a_{1}+a_{2}}{2}. \end{aligned}$$

Application of the following three steps to the state space representation (48) yields the fundamental dynamics (17):

S4 Approximations through 3rd order Taylor polynomials:
  $$\begin{aligned} \sin x \approx x - \frac{x^3}{3!}, \cos x \approx 1 - \frac{x^2}{2!} \end{aligned}$$
S5 Use of trigonometric identities:
  $$\begin{aligned} \sin ^2 x+ \cos ^2 x= & {} 1, \sin (2x)=2 \sin x \cos x,\\ \cos (2x)= & {} \cos ^2 x - \sin ^2 x \end{aligned}$$
  And deduced from above:
  $$\begin{aligned} \sin ^2 x= \frac{1}{2} - \frac{1}{2} \cos (2x), \cos ^2 x= \frac{1}{2} + \frac{1}{2} \cos (2x) \end{aligned}$$
S6 Neglect of higher harmonics, e.g. $\sin (2x) \approx 0$, $\cos (4x) \approx ~0$

Use of the actual natural frequency for normalization of the phase space ${\varOmega =\omega }$ reduces the error caused by the approximations ${\vartheta _E \approx \vartheta _r}$.

**Phase dynamics** $$\dot{\varphi }$$:

49 $$\begin{aligned} f_{\mathrm {p},1}\mathop {=}\limits ^{\varOmega =\omega }&\omega \sin ^2\varphi +\frac{\omega _0^2}{\omega \vartheta _{r}}\cos \varphi \sin (\vartheta _{r}\cos \varphi )\nonumber \\ \mathop {\approx }\limits ^{\mathrm {S}4}&{\omega \sin ^2\varphi + \frac{\omega _0^2}{\omega \vartheta _{r}}\cos \varphi \bigg (\vartheta _{r} \cos \varphi - \frac{\vartheta _r^3 \cos ^3\varphi }{6}\bigg )}\nonumber \\ \mathop {=}\limits ^{\mathrm {S5}}&{\omega \left( \frac{1}{2} - \frac{1}{2} \cos (2 \varphi )\right) + \frac{\omega _0^2}{\omega }\left[ \left( \frac{1}{2} + \frac{1}{2} \cos (2\varphi )\right) \right. } \nonumber \\&{\left. - \frac{\vartheta _r^2}{6} \left( \frac{1}{2} + \frac{1}{2} \cos (2\varphi ) \right) ^2 \right] }\nonumber \\ \mathop {\approx }\limits ^{\mathrm {S5},6}&{\omega \left( \frac{1}{2}\right) + \frac{\omega _0^2}{\omega }\left[ \left( \frac{1}{2}\right) - \frac{\vartheta _r^2}{6} \left( \frac{1}{4} + \frac{1}{2} \cos (2\varphi ) \; \right. \right. } \nonumber \\&{\left. \left. +\frac{1}{4} \left( \frac{1}{2}+ \frac{1}{2}\cos (4\varphi )\right) \right) \right] }\nonumber \\ \mathop {\approx }\limits ^{\mathrm {S6}}&{\frac{1}{2} \omega + \frac{\omega _0^2}{\omega }\left[ \frac{1}{2} - \frac{\vartheta _r^2}{6} \left( \frac{1}{4} + \frac{1}{8} \right) \right] }\nonumber \\ \mathop {=}\limits ^{}&\frac{1}{2} \omega + \frac{1}{2 \omega } \omega _0^2 \left( 1- \frac{1}{2} \left( \frac{\vartheta _r}{2}\right) ^2 \right) \nonumber \\ \mathop {\approx }\limits ^{\mathrm {S}4^{-1}}&\frac{1}{2} \omega + \frac{1}{2 \omega } \omega _0^2 \left( \cos \left( \frac{\vartheta _r}{2}\right) \right) \nonumber \\ \mathop {\approx }\limits ^{\omega _g}&{\frac{1}{2} \omega + \frac{1}{2 \omega } \omega _g^2 } \mathop {\approx }\limits ^{\omega _g \approx \omega } \omega , \end{aligned}$$

with “$\mathrm {S}4^{-1}$” indicating application of the 3rd order Taylor approximation in reverse direction and insertion of the geometric mean approximation (9) with $\vartheta _E\approx \vartheta _r$ in the last step. For $^ag_{\mathrm {p},1}$, the approximation steps S4 to S6 as detailed in (49) yield $^ag_{\mathrm {p},1} \approx 0$, independent of the actuation terms $A_{{\ddot{r}}}$ and $A_t$. Consequently, the phase dynamics for the abstract cart- and torque-pendulums result in $\dot{\varphi }\approx \omega$.

**Energy dynamics** $\dot{\vartheta }_r$: Similar to $^ag_{\mathrm {p},1}$, the approximation steps S4 to S6 result in $f_{\mathrm {p},2} \approx 0$. The remaining term $^ag_{\mathrm {p},2}$ simplifies for the abstract cart-pendulum to

50 $$\begin{aligned} {}^ag_{\mathrm {p},2,{\ddot{r}}}\mathop {=}\limits ^{\varOmega =\omega }&\frac{\omega _0^2 \omega }{g}\sin ^2\varphi \cos (\vartheta _{r}\cos \varphi )\nonumber \\ \mathop {\approx }\limits ^{\mathrm {S}4}&{\frac{\omega _0^2 \omega }{g}\sin ^2\varphi \left( 1- \frac{1}{2} \vartheta _r^2 \cos ^2\varphi \right) } \nonumber \\ \mathop {=}\limits ^{\mathrm {S5}}&\frac{\omega _0^2 \omega }{g} \left( \frac{1}{2}- \frac{1}{2} \cos (2\varphi ) \right) \nonumber \\&\left[ 1 - \frac{\vartheta _r^2}{2} \left( \frac{1}{2}+ \frac{1}{2} \cos (2\varphi ) \right) \right] \nonumber \\ \mathop {=}\limits ^{}&\frac{\omega _0^2 \omega }{g} \left[ \left( \frac{1}{2}- \frac{1}{2} \cos (2\varphi ) \right) \right. \nonumber \\&\left. - \frac{\vartheta _r^2}{2} \left( \frac{1}{4}- \frac{1}{4} \cos ^2(2\varphi ) \right) \right] \nonumber \\ \mathop {\approx }\limits ^{\mathrm {S5,6}}&{\frac{\omega _0^2 \omega }{g} \left[ \left( \frac{1}{2} \right) - \frac{\vartheta _r^2}{2} \left( \frac{1}{4}- \frac{1}{8} \right) \right] } \nonumber \\ \mathop {=}\limits ^{}&\frac{\omega _0^2 \omega }{2g} \left( 1 - \frac{1}{2} \left( \frac{\vartheta _r}{2} \right) ^2 \right) \nonumber \\ \mathop {\approx }\limits ^{\mathrm {S}4^{-1}}&\frac{\omega }{2g}\omega _0^2 \left( \cos \left( \frac{\vartheta _r}{2}\right) \right) \nonumber \\ \mathop {\approx }\limits ^{\omega _g}&{\frac{\omega }{2g}\omega _g^2} \mathop {\approx }\limits ^{\omega _g \approx \omega } \frac{1}{2g}\omega ^3 = B_{{\ddot{r}}}. \end{aligned}$$

As for (49), we applied a reverse 3rd order Taylor approximation ($\mathrm {S}4^{-1}$) and inserted the geometric mean approximation of the natural frequency $\omega _{{\mathrm {g}}}$ in (9).

For the abstract torque-pendulum we get

51 $$\begin{aligned} {}^ag_{\mathrm {p},2,t}&=\frac{1}{\omega I_\vartheta }\sin ^2\varphi \nonumber \\&\mathop {\approx }\limits ^{\mathrm {S}5,6}\frac{1}{2\omega I_\vartheta } =: B_t. \end{aligned}$$

Thus, the fundamental energy dynamics linearly depends on the amplitude factors ${\dot{\vartheta }}_r \approx B \frac{a_{1}+a_{2}}{2}$. The result are the fundamental dynamics in (17).

## Stability of the $\omega$-Estimation

For an approximately constant natural frequency $\omega$ we have $\varphi (t)= \omega t$, where we set $\varphi (t=0)=0$ without loss of generality (see (11)). This yields the modified state transformations $\vartheta =\vartheta _r \cos (\omega t)$ and ${\dot{\vartheta }}=-\vartheta _r \omega \sin (\omega t)$ compared to (16), and the phase computation results in

52 $$\begin{aligned} \varphi = {{\mathrm{atan2}}} \left( -\frac{{\dot{\vartheta }}}{\hat{\omega }} , \vartheta \right) = {{\mathrm{atan2}}} \left( {\frac{\omega }{\hat{\omega }}\sin (\omega t)} , \cos (\omega t) \right) , \end{aligned}$$

which is independent of $\vartheta _r$. Consequently, the natural frequency estimation in Fig. 6 has one input, the natural frequency $\omega$, and one output, the estimate ${\hat{\omega }}$. Note that we assume $\omega$ to be known only for the stability analysis, but not for the implementation displayed in Fig. 6.

In a next step, we derive the estimation dynamics in terms of its input $\omega$ and output ${\hat{\omega }}$. Differentiation of (52) with respect to time yields

53 $$\begin{aligned} {\dot{\varphi }}&\mathop {=}\limits ^{} {\frac{ \frac{\omega ^2}{{\hat{\omega }}} \sin ^2(\omega t) + \cos (\omega t) \left( \frac{\omega ^2}{{\hat{\omega }}} \cos (\omega t) - \frac{\omega }{{\hat{\omega }}^2} \dot{\hat{\omega }} \sin (\omega t) \right) }{\frac{\omega ^2}{{\hat{\omega }}^2} \sin ^2(\omega t) + \cos ^2(\omega t)}}\nonumber \\&\mathop {=}\limits ^{\cos ^{-2}(\omega t)} {\frac{\frac{\omega ^2}{{\hat{\omega }}} \tan ^2(\omega t)+ \frac{\omega ^2}{{\hat{\omega }}} -\frac{\omega }{{\hat{\omega }}^2}\tan (\omega t)\;\dot{{\hat{\omega }}}}{1+\frac{\omega ^2}{{\hat{\omega }}^2}\tan ^2(\omega t) }.} \end{aligned}$$

Transformation of (19) into time domain yields

54 $$\begin{aligned} \dot{\hat{\omega }}=-\frac{1}{T_\omega }(\hat{\omega }-\dot{\varphi }). \end{aligned}$$

Insertion of (54) solved for ${\dot{\varphi }}$ into (53), followed by some rearrangements yields the $\omega$-estimation dynamics

55 $$\begin{aligned} \dot{{\hat{\omega }}}= & {} \frac{{\hat{\omega }}\omega ^2-{\hat{\omega }}^3}{T_\omega {\hat{\omega }}^2{+}\omega \tan (\omega t)+T_\omega \omega ^2\tan ^2(\omega t)}. \end{aligned}$$

Because $\omega$ is bounded and constant, it suffices to show stability of the estimation error dynamics $\dot{\tilde{\omega }} = \dot{\hat{\omega }}-{\dot{\omega }} = \dot{\hat{\omega }}$. As Lyapunov function we choose

56 $$\begin{aligned} V=\frac{1}{2}\left( {\hat{\omega }}-\omega \right) ^2 \end{aligned}$$

with time derivative

57 $$\begin{aligned} {\dot{V}}= & {} \frac{-{\hat{\omega }}({\hat{\omega }}-\omega )^2({\hat{\omega }} +\omega )}{T_\omega \omega ^2\tan ^2(\omega t) {+}\omega \tan (\omega t)+ T_\omega {\hat{\omega }}^2}. \end{aligned}$$

For the numerator of (57) holds that $-{\hat{\omega }}({\hat{\omega }}-\omega )^2({\hat{\omega }}+\omega ) \le 0$ if ${{\mathrm{sgn}}}(\omega )={{\mathrm{sgn}}}({\hat{\omega }})$. The denominator is a quadratic function of $\tan (\omega t)$, with $-\infty< \tan (\omega t)<\infty$. From $T_\omega \omega ^2 > 0$ we deduce that the denominator with $\tan (\omega t) = x$ is a convex parabola. Therefore, we have a positive denominator, if the discriminant $\mathcal {D}$ is negative, i.e.

58 $$\begin{aligned} \mathcal {D} =\omega ^2-4 T_\omega \omega ^2 T_\omega {\hat{\omega }}^2<0 \;\quad \Rightarrow \quad T_\omega > \frac{1}{2 {\hat{\omega }}}. \end{aligned}$$

Condition (58) depends on the natural frequency estimate ${\hat{\omega }}$, which varies over time. Because we are estimating the natural frequency of a pendulum under the influence of gravity, only positive values are physically plausible $\omega >0$. For $T_\omega > \frac{1}{2 {\hat{\omega }}(t=0)}$ and $\omega \ne {\hat{\omega }}(t=0)>0$, we have ${{\mathrm{sgn}}}(\omega )={{\mathrm{sgn}}}({\hat{\omega }}(t=0))$ and ${\dot{V}} (t=0) < 0$ and ${\hat{\omega }}$ initially approaches $\omega$. If further $T_\omega > \frac{1}{2 \omega }$, ${\dot{V}}(t \ge 0) < 0$ as long as $\omega \ne {\hat{\omega }}$ and (58) can be rewritten as

59 $$\begin{aligned} {T_\omega > \max \left( \frac{1}{2 {\hat{\omega }}(t=0)},\frac{1}{2 \omega }\right) .} \end{aligned}$$

Thus, if (59) holds, the $\omega$-estimation is asymptotically stable under the fundamental dynamics assumption. This proves convergence of the estimate $\hat{\omega }$ to the true value $\omega$ for a linearly oscillating pendulum.

## Transfer Functions of Leader–Follower Structures

Rearrangement of the block diagram in Fig. 7 leads to the block diagram displayed in Fig. 19. The highlighted intermediate transfer function $G^\mathrm {fi}_1$ is

60 $$\begin{aligned} {G^\mathrm {fi}_1=\frac{\frac{1}{s}}{1-\frac{1}{s} \varGamma _{\mathcal {F}} \frac{B}{{\hat{B}}_{\mathcal {F}}} \frac{s}{T_{\mathcal {F}} s +1} }.} \end{aligned}$$

Based on (60) the reference input transfer function $\vartheta _r(s)=G^{\mathrm {fi}}(s)\theta _E^\mathrm {d}(s)$ results in (27).

For the computation of the relative follower contribution $\varGamma _{\mathcal {F}}$, consider the block diagram rearrangement in Fig. 20. From Fig. 20 with

61 $$\begin{aligned} {G^\mathrm {fi}_2=\frac{1}{1-\varGamma ^\mathrm {d}_{\mathcal {F}} \frac{B}{{\hat{B}}_{\mathcal {F}}} \frac{1}{T_{\mathcal {F}} s +1} },} \end{aligned}$$

we can compute the transfer function which yields the amount of energy the leader contributes $\vartheta _{r{\mathcal {L}}}(s)$ based on the reference input $\theta _E^\mathrm {d}(s)$

62 $$\begin{aligned} G^\mathrm {fi}_{\mathcal {L}}=\frac{\varGamma ^\mathrm {d}_{\mathcal {L}} K_\mathrm {d} \frac{B}{{\hat{B}}_{\mathcal {L}}} \left( s+\frac{1}{T_{\mathcal {F}}}-\varGamma ^\mathrm {d}_{\mathcal {F}} \frac{B}{{\hat{B}}_{\mathcal {F}}} \frac{1}{T_{\mathcal {F}}}\right) }{s^2+\left( \frac{1}{T_{\mathcal {F}}} -\varGamma ^\mathrm {d}_{\mathcal {F}} \frac{B}{{\hat{B}}_{\mathcal {F}}}\frac{1}{T_{\mathcal {F}}} + \varGamma ^\mathrm {d}_{\mathcal {L}} K_\mathrm {d} \frac{B}{{\hat{B}}_{\mathcal {L}}}\right) s + \varGamma ^\mathrm {d}_{\mathcal {L}} K_\mathrm {d} \frac{B}{{\hat{B}}_{\mathcal {L}}} \frac{1}{T_{\mathcal {F}}}}. \nonumber \\ \end{aligned}$$

From $\vartheta _{r{\mathcal {F}}}(s)= \vartheta _r(s) - \vartheta _{r{\mathcal {L}}}(s) = G^{\mathrm {fi}}(s)\theta _E^\mathrm {d}(s) - G^\mathrm {fi}_{\mathcal {L}}(s) \theta _E^\mathrm {d}(s)$ with (27) and (62) we get $G^\mathrm {fi}_{\mathcal {F}}(s) = G^{\mathrm {fi}}(s)- G^\mathrm {fi}_{\mathcal {L}}(s)$ in (28).

## Footnotes

1 We furthermore assume that a low-level robot control has access to the end effector position.

2 These oscillations can be damped through application of the controller presented in Sect. 5 in *z*-direction, as shown in [12] for the non-adaptive control approach of [13].

3 In contrast to the t-pendulum and the afa-system, the simple pendulum approximations are modeled as rigid and can thus reach oscillation amplitudes beyond $90^{\circ }$. In order to challenge our approach, we command $\theta _E^\mathrm {d}>90^{\circ }$ here.

4 The *KUKA LWR 4+* can handle higher loads, if operated close to its singularities. However, joint velocity limits restrict the end effector velocity. As we are interested in a proof of concept of the proposed approach independent of the robotic platform used, we refrained from optimizing the robotic setup for higher loads and velocities.

5 Parameters were set once based on theoretic results ($T_\omega$, ${\hat{\omega }}(t=0)$) or according to their physical meaning, i.e. they resemble filter coefficients ($T_{\mathcal {F}}$, $D_{\mathcal {F}}$, $c_0$, $\zeta$, $l_1$), the human-like arm dynamics of the afa-system ($I_{\rho /\psi }$, $d_{\rho /\psi }$, $k_\psi$, $m_\mathrm {a}$, $l_\mathrm {a}$) or define desired leader/follower behavior ($K_\mathrm {d}$, $\varGamma _{{\mathcal {L}}/{\mathcal {F}}}$).
